# *Salvia miltiorrhiza* Bunge (Danshen) and Bioactive Compound Tanshinone IIA Alleviates Cisplatin-Induced Acute Kidney Injury Through Regulating PXR/NF-κB Signaling

**DOI:** 10.3389/fphar.2022.860383

**Published:** 2022-03-24

**Authors:** Jing-Yun Dou, Min Zhang, Huan Cen, Yi-Qin Chen, Yi-Fan Wu, Fuhua Lu, Jiuyao Zhou, Xu-Sheng Liu, Yue-Yu Gu

**Affiliations:** ^1^ Department of Nephrology, Guangdong Provincial Hospital of Chinese Medicine, The Second Affiliated Hospital of Guangzhou University of Chinese Medicine, Guangzhou, China; ^2^ Department of Ultrasound, The Second Affiliated Hospital of Guangzhou University of Chinese Medicine, Guangzhou, China; ^3^ Department of Pharmacology, School of Pharmaceutical Sciences, Guangzhou University of Chinese Medicine, Guangzhou, China

**Keywords:** acute kidney injury, pregnane X receptor, NF-κB, *Salvia miltiorrhiza*, tanshinone IIA, renal inflammation

## Abstract

**Objective:** The present study aims to provide evidence on the potential protective role of *Salvia miltiorrhiza* Bunge (Danshen) and its bioactive compound Tanshinone IIA (TanIIA) in AKI and to reveal the specific regulatory function of PXR/NF-κB signaling in AKI-induced renal inflammation.

**Methods:** A network pharmacological analysis was used to study target genes and regulatory networks in the treatment of *Salvia miltiorrhiza* on AKI. Further experiments with *in vivo* AKI mouse model and *in vitro* studies were applied to investigate the renal protective effect of TanIIA in AKI. The mechanisms of TanIIA regulating PXR/NF-κB signaling in renal inflammation were also studied.

**Results:** Network pharmacology had suggested the nuclear receptor family as new therapeutic targets of *Salvia miltiorrhiza* in AKI treatment. The *in vivo* studies had demonstrated that TanIIA improved renal function and inflammation by reducing necrosis and promoting the proliferation of tubular epithelial cells. Improved renal arterial perfusion in AKI mice with TanIIA treatment was also recorded by ultrasonography. *In vitro* studies had shown that TanIIA ameliorated renal inflammation by activating the PXR while inhibiting PXR-mediated NF-κB signaling. The results had suggested a role of PXR activation against AKI-induced renal inflammation.

**Conclusion:** *Salvia miltiorrhiza* Bunge (Danshen) may protect the kidneys against AKI by regulating nuclear receptors. TanIIA improved cell necrosis proliferation and reduced renal inflammation by upregulating the expression of the PXR and inhibiting NF-κB signaling in a PXR-dependent manner. The PXR may be a potential therapeutic target for AKI treatment.

## Graphical Abstract

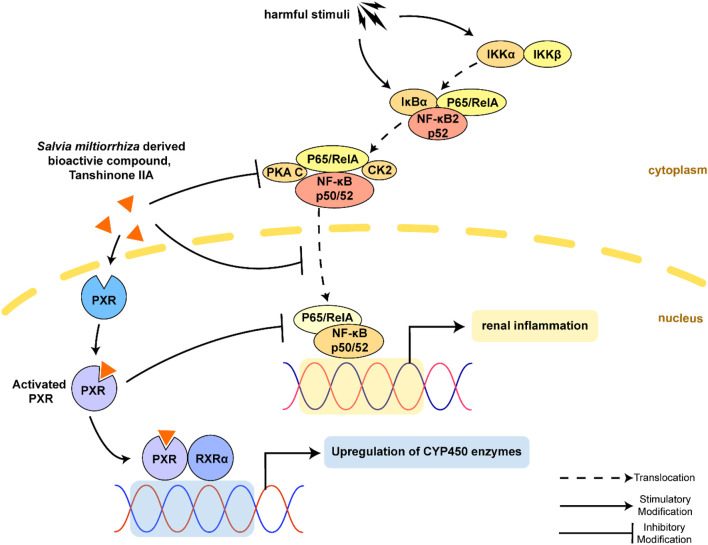

## Introduction

Acute kidney injury (AKI) involves a rapid decline in renal function within hours to days. It is a global burden with a high mortality rate that exceeds breast cancer, heart failure, or diabetes so far (Kellum et al., 2021). Approximately 15% of patients in hospital, and more than 57% of patients in the intensive care unit (ICU) develop AKI. (Hoste et al., 2015; Scholz et al., 2021). Nowadays, treatment and therapies for AKI vary, depending on the cause of kidney injuries and preventing complications until the kidneys recover. The initial renal insults may induce the infiltration of inflammatory leukocytes within the glomerulus and tubulointerstitial area. Unresolved renal inflammation can impair the function of the nephrons and lead to a reduction in the estimated glomerular filtration rate (eGFR) (Gu et al., 2021). We are now clear that renal inflammation is the central pathogenic mechanism that drives AKI. However, the mechanisms of renal inflammation are not fully elaborated, and practical strategies and therapeutic drugs are still limited.

The pregnane X receptor (PXR, NR1I2) is a well-known member of the nuclear receptor superfamily (He et al., 2017). The PXR functions as a ligand-activated transcription factor to upregulate the activity and expression of drug-metabolizing enzymes (i.e., phase I oxidative enzymes: cytochrome P450 (CYP450) enzymes), conjugating enzymes (phase II enzyme such as glutathione S-transferase, GST), and transporters (phase III transport uptake and ATP-dependent efflux pump such as multidrug resistance protein 1, MDR1). These enzymes are key mediators in regulating detoxification and clearance of xenobiotics. The PXR contains a DNA-binding domain (DBD) and a ligand-binding domain (LBD) connected by a hinge region. The LBD is a mostly hydrophobic flexible pocket for a wide range of drugs and compounds. The PXR then forms a transcriptionally active complex with retinoid X receptor α (RXRα) and binds to the DNA response elements to control gene expression associated with xenobiotic and endobiotic metabolism. The PXR can be activated by a wide range of endogenous and exogenous ligands such as hormone metabolites, bile acids, and drugs (Hogle et al., 2018); it also acts as a key player in inflammation, energy metabolism, and endocrine homeostasis (Oladimeji and Chen, 2018).

In humans, the PXR is mainly expressed in the liver, intestine, and kidneys. As for the kidneys, the proximal tubular epithelial cells (TECs) express the PXR at a relatively high level. A variety of studies have highlighted the significant role of the PXR in renal toxicity, diabetic nephropathy, and chronic kidney disease (CKD) (Velenosi et al., 2014; Lee et al., 2018; Watanabe et al., 2018). Nuclear factor κB (NF-κB) is a protein complex that controls an extensive array of genes in immune response evoked by harmful cellular stimuli. The NF-κB signaling acts as a significant determinant for the progression of renal inflammatory pathogenesis of AKI (Markó et al., 2016; Song et al., 2019). Interestingly, previous studies on inflammatory bowel disease and liver inflammation had demonstrated the suppressing effect of the PXR on NF-κB activity (Deuring et al., 2019; Okamura et al., 2020), suggesting the anti-inflammatory effect of the PXR. However, the mechanisms of the PXR on NF-κB-induced renal inflammation and therapies are still lacking.

*Salvia miltiorrhiza* Bunge (Danshen) is a perennial plant that is highly valued for its roots in traditional Chinese medicine. Tanshinones are critical lipophilic diterpenoids from Danshen extracts. These secondary metabolites are essential resources derived from medicinal plants (Ma et al., 2015). Among tanshinones, TanIIA is the most abundant compound that exerts anti-inflammatory effects in various diseases (Guo et al., 2020). Moreover, TanIIA was reported to be an PXR activator and induce the transcriptional level of cytochrome P450 3A4 (CYP3A4) (Yu et al., 2009; Liu et al., 2011). Mechanistic studies have revealed the anti-inflammatory effect of TanIIA through PXR activation (Zhang et al., 2015a; Zhang et al., 2015b; Zhu et al., 2017).

Based on previous findings, we hypothesized that *Salvia miltiorrhiza* and its active component, TanIIA, may protect the kidneys against AKI by regulating the PXR/NF-κB signaling in renal inflammation. A network pharmacological analysis was applied to investigate target genes and regulatory networks modulated by *Salvia miltiorrhiza*. Moreover, we elucidated the protective effects and underlying mechanisms of TanIIA on AKI *in vivo* and *in vitro*. A molecular docking study was also applied to reveal the possible drug–protein interaction mode of TanIIA and PXR. The present study provides novel aspects on the anti-inflammation effect of *Salvia miltiorrhiza* and reveals a role of the PXR/NF-κB signaling pathway in AKI. PXR may serve as one of the promising therapeutic targets against AKI-induced inflammation.

## Materials and Methods

### Prediction of *Salvia miltiorrhiza*-Associated Target Genes, Their Intersection on AKI, and Analysis on Gene Ontology (GO) and Enrichment KEGG pathways

A network pharmacological analysis was applied to study related genes in treating *Salvia miltiorrhiza* on AKI. We searched the keyword “Danshen” in the Traditional Chinese Medicine Systems Pharmacology Database and Analysis Platform (TCMSP http://tcmspw.com/tcmsp.php) (Ru et al., 2014) to obtain active ingredients and their ADME (absorption, distribution, metabolism, and excretion) information. OB (oral bioavailability) and DL (drug-likeness) were recorded. The criteria of OB ≥ 30% and DL ≥ 0.18 suggest that the ingredients with good absorption into blood were screened as potential active ingredients. Then, the corresponding target proteins of the active ingredients were collected from the TCMSP database, and corresponding target genes were analyzed *via* String database (Von Mering et al., 2003) UniProt databases (The UniProt Consortium, 2018). The target genes of AKI were obtained from the GeneCards (https://www.genecards.org/), OMIM (https://omim.org/), and DisGeNET database (https://www.disgenet.org/). We used the Venn online analysis tool (https://www.omicshare.com/) to study the intersection target genes of *Salvia miltiorrhiza* and AKI. These genes are considered potential targets of *Salvia miltiorrhiza* for treating AKI. With those intersection target genes, we performed GO and KEGG pathway enrichment analyses by using the ClueGO plug-in of Cytoscape 3.6.0. The screening was based on *p* < 0.01 and a κ score ≥ 0.53 to visualize the results of GO and KEGG enrichment (Bindea et al., 2009). A flowchart of network pharmacology study is shown in Supplementary Figure S3. The original data in the network pharmacological analysis are available on Figshare (https://doi.org/10.6084/m9.figshare.17895506.v1)

### Animal Model of Cisplatin-Induced AKI and TanIIA Treatment

TanIIA and cisplatin were HPLC-purified products purchased from Sigma-Aldrich. The chemical structure of TanIIA was constructed by ChemOffice (CambridgeSoft, Cambridge, MA) as shown in Figure 7A. TanIIA was dissolved in 150 μl saline with 0.01% DMSO. Male C57BL/6 mice at eight weeks of age (weighting 20–25 g) were housed under 23 ± 2°C, 55 ± 5% humidity, and 12/12-hour light/dark cycle environment with free access to water and food. The number of mice was calculated and assigned to groups following an allocation process (Charan and Kantharia, 2013; Bespalov et al., 2020). The mice were divided into 4 groups (*n* = 5): normal control (CTRL), AKI model, AKI+TanIIA12.5 (12.5 mg/kg/day, i.p.) low-dose treatment, and AKI+TanIIA25 (25 mg/kg/day, i.p.) high-dose treatment group. The doses were chosen based on previous publications (Jiang et al., 2015; Zhang et al., 2020). The AKI treatment groups received a single cisplatin injection (20 mg/kg, i.p.). CTRL received the same volume of control saline intraperitoneally. The body weight of all mice was recorded before and after 72 h of the cisplatin injection. The mice were killed on AKI day 3. Serum, kidneys, and other organs were collected after ultrasonic assessment for renal blood flow. The experimental protocol was approved by the Institutional Animal Care and Use Committee of Guangdong Provincial Hospital of Chinese Medicine (Approval No. 2020020).

### Arterial Ultrasonography and Renal Function

Renal ultrasonography was performed to visualize renal blood vessels using a high-frequency ultrasound imaging system (Vevo 2100, Visual Sonics Inc., Toronto, Canada) as previously described (Okumura et al., 2018). All measurements were done on the right abdomen. Peak systolic and end-diastolic blood flow velocities (mm/s) were measured in the renal artery to obtain the pulsatility index (PI). An experienced technician who was blinded to the study protocol repeated the measurement three times. According to the manufacturer’s instructions, serum creatinine was measured with commercial kits (C011-2-1, Nanjing Jiancheng Bioengineering Institute, China).

### Cell Culture and Cytotoxicity Assay

Human proximal tubular cell line HK-2 was purchased from American Type Culture Collection (CRL-2190, ATCC, VA, United States). HK-2 cells were cultured in DMEM/F12 medium supplemented with 10% (v/v) FBS (Gbico, United States) under a humidified atmosphere with 5% CO_2_ at 37°C. Cells were trypsinized and harvested when the confluence reached 80–90%.

Cytotoxicity activity was assayed by using a Cell Counting Kit-8 (CCK-8) (Dojindo, Japan) according to the manufacturer’s instructions. Dimethyl sulfoxide (DMSO) was used as the vehicle and did not exceed 0.1%. Each cell line would be seeded into a new flask and treated with 5% DMEM/F12 medium with DMSO (0.1% v/v) and TanIIA at different concentrations. HK-2 cells were, respectively, seeded into 96-well plates at a density of 5 × 10^3^ cells/well, and cultured in the DMEM/F12 with a low FBS concentration for 24 h. Then they were divided into control groups, DMEM/F12 with DMSO (0.1% v/v) (100 μl/well) and TanIIA group at various final concentrations of 3.125, 6.25, 12.5, 25, 50, and 100 μM (100 μl/well). After incubating for 6, 12, and 24 h, the medium was replaced with 10 μl 10% CCK-8 solution dissolved in 90 μl PBS, and all the cells were incubated for another 1h at 37°C in the dark. Absorbance at 450 nm was measured using a Sunrise™ Microplate Reader (TECAN, Switzerland). Each concentration was repeated five times in the same plate, and all experiments were performed at least three times. The percentage of cell viability was normalized to control group cells to calculate the inhibition rate:

$$Inhibition\;rate\;\left(IR\right)\;\left(\%\right)=1-\frac{Sample\;solution\;OD\;value}{Control\;OD\;value}\times 100\%.$$

IC_50_ was calculated with the Excel add-in tool ED50plus (created by Mario H. Vargas, MD).

### Gene Silencing of PXR

In total, 1 × 10^6^ HK-2 cells were transfected with the hPXR small-interfering RNA (siRNA) or the control siRNA (Santa Cruz Biotechnology, CA, United States) using Lipofectamine 3000 transfection reagent (Thermo Fisher Scientific Inc., United States) according to the manufacturer’s instructions. The control siRNA was used as a negative control. RIF (rifampin) (Sigma-Aldrich, United States) (10 µM) was used as the positive control. Cells were treated with DMSO (0.1% v/v), RIF (10 µM), and TanIIA (5 µM) and were stimulated with/without TNF-α (20 ng/ml) for 24 h. The cells were then subjected to the NF-κB luciferase reporter assay.

### PXR Transactivation Reporter Assay and PXR-Mediated NF-κB Reporter Assay

The pSG5-hPXR expression plasmids and pGL3-CYP3A4-XREM luciferase reporter construct, containing the basal promoter (−10466/+53) with the proximal PXR response element (ER6) and the distal xenobiotic responsive enhancer module (XREM, −7836/−7208) of the CYP3A4 gene 5′-flanking regions inserted to pGL3-basic reporter vector, were kind gifts from Dr. Sridhar Mani (Albert Einstein College of Medicine, Bronx, NY, United States) (Huang et al., 2007). The pRL-TK *Rotylenchulus reniformis* control vector was purchased from Promega, Madison, United States.

HK-2 cells were seeded at 60–70% confluence into opaque 96-well plates at a density of 1 × 10^5^ cells/well in DMEM/F12 containing 10% FBS and cultured for 12 h. The cells were transiently transfected with pGL3-CYP3A4-XREM, pSG5-hPXR nuclear receptor expression vector, and pRL-TK control vector in a particular ratio with Lipofectamine 3000 transfection reagent according to the manufacturer’s instructions. After 6 h of incubation, the transfected HK-2 cells were exposed to TanIIA at 1, 2.5, 5, and 10 μM; RIF (10 μM); and control (0.1% DMSO) for an additional 24 h. Following the treatments, HK-2 cells were rinsed with PBS, and the luciferase activities were determined using a Dual-Luciferase Reporter Assay System (Promega, Madison, United States) and an Infinite F500 multimode microplate reader (TECAN, Switzerland).

HK-2 cells were transiently transfected with pGL4.32 [luc2P/NF-κB-RE/Hygro] NF-κB reporter, pCMV6-entry or pCMV6-XL4-hPXR, and pRL-TK (Promega, Madison, United States) with Lipofectamine 3000 transfection reagent according to the manufacturer’s instructions. After overnight transfection, the cells were treated as control (0.1% DMSO), TanIIA (5 μM), and RIF (10 μM) for 24 h, and additional incubation with/without TNF-α (20 ng/ml) for another 24 h. A standard dual luciferase assay was performed on cell lysates and detected using an Infinite F500 multimode microplate reader.

Transfection efficiency was expressed as fold induction of firefly to *Renilla* luciferase activities relative to control. Each concentration of the test substances was repeated five times in the same plate, and the transfection experiments were performed at least three times. Data are expressed as mean ± SEM from three independent experiments.

### Histology and Immunohistochemistry

Tissues were fixed with 4% buffered paraformaldehyde for 48 h, and they were cut into 3-μm paraffin sections for hematoxylin and eosin (HE), periodic acid–Schiff (PAS) and Masson’s trichrome staining. The morphology of renal tubules with prominent necrosis were recorded. Patchy or diffuse denudation with loss of brush border detachment of cells with intratubular cast formation was identified. The degree of tubular injury was scored as score 0: no tubular injury; score 1: ≤10% tubules injured; score 2: 11–25% tubules injured; score 3: 26–50% tubules injured; score 4: 51–74% tubules injured; and score 5: ≥75% tubules injured.

Immunohistochemistry (IHC) sections were de-waxed and proceeded by a microwave-based antigen retrieval technique following previous procedures (Gu et al., 2018). Antibodies used in IHC included PCNA Rabbit mAb IKKβ Rabbit mAb (Cell Signaling Technology, MA, United States), anti-NF-κB p105/p50 Rabbit mAb (Abcam, MA, United States). The kidney sections were stained with primary and secondary antibodies. The sections were developed with diaminobenzidine (DAB) to produce a brown color. The percentage of positive staining areas of PCNA, NF-κB p105/p50, and IKKβ expression was measured by ImageJ software (NIH, Bethesda, MD, United States).

### Immunofluorescence Staining

HK-2 cells were seeded, incubated, and treated with 0.1% DMSO, TanIIA (5 µM), and/or TNF-α (20 ng/ml) for 24 h. Cells were fixed with 4% paraformaldehyde for 30 min, permeabilized with 0.5% Triton X-100 for 20 min, and blocked with 5% BSA for 30 min at room temperature. Primary antibodies against NF-κB p65 (1:100, Cell Signaling Technology, MA, United States) were incubated with cells at 4°C overnight. After washing with PBS three times, an FITC-conjugated secondary antibody was added for 60 min at room temperature. All slides were mounted with DAPI-containing mounting medium and then analyzed with a fluorescence microscope (Leica Microsystems, Wetzlar, Germany)

### ELISA

Mouse serum was collected and centrifuged at 3,000 rpm for 15 min at 4°C. The level of neutrophil gelatinase-associated lipocalin (NGAL), IL-6, and TNF-α in blood was detected by using ELISA kits (R&D Systems Inc., Minneapolis, United States), following the manufacturer’s instructions.

### RNA Extraction and Real-Time PCR

Total RNA was isolated from the kidney tissue with the TRIzol reagent (Thermo Fisher Scientific Inc., United States) according to the manufacturer’s protocol. Total RNA was quantified by the absorbance ratio (260/280 nm) and reverse-transcribed into cDNA using PrimeScript™ RT reagent (Takara Bio Inc., Japan). All the PCR reactions were carried out using SYBR® Premix EX Taq™ II Kit (Takara Bio Inc., Japan) in 96-well optical plates on an ABI ViiA™ 7 PCR System (Applied Biosystems, Thermo Fisher Scientific, United States). The quantity of each transcript was calculated as described in the instrument manual and normalized to the housekeeping gene β-actin. The ratio of target to β-actin was calculated as ΔC_t=_C_t_ (target)-C_t_ (β-actin), ratio (target) = 2^−ΔΔCt^. The primers used for real-time PCR are listed in Supplementary Table S5.

### Western Blot Analysis

The proteins from renal cortical tissues were extracted with RIPA lysis buffer. The samples were subjected to Western blot analysis with primary antibodies against phospho-NF-κB p65, NF-κB p65, IKKβ, NF-κB p105/p50 (Cell Signaling Technology, MA, United States), RXRα (Abcam, MA, United States), PXR, and GAPDH (Santa Cruz Biotechnology, CA, United States) overnight at 4°C and subsequently incubated with the conjugated secondary antibody of horseradish peroxidase-labeled anti-rabbit IgG or anti-mouse IgG (Cell Signaling Technology, MA, United States) at room temperature. Target protein expression was detected by using an Image Lab System (Bio-Rad Laboratories Inc., Hercules, CA, United States) and analyzed with ImageJ software.

### Molecular Docking

The ligand and the receptor recognize each other and bind together by geometry and energy matching. Molecular docking provides new insights into the ligand–receptor interactions and structural features of the two molecules within the active site. To predict the protein–ligand interaction, we performed the CDOCKER algorithm and Libdock module in Discovery Studio (DS) 2.5 (Accelrys Software Inc., San Diego, United States) to evaluate the potential interaction of ligands and the proteins, respectively. The 3D crystal structure of TanIIA (CID: 164676) was obtained from the PubChemProject (http://pubchem.ncbi.nlm.nih.gov/compound/). The receptor PXR was selected from Protein Data Bank (PDB) (http://www.rcsb.org/pdb/). The program was run by using a localhost9943 server on Microsoft Windows 7.

The calculation of root mean square deviation (RMSD) was carried out. RMSD indicates the reliability of the results from the above modules. Docking procedures on DS 2.5 were as follows: the water molecules in the targeted protein PXR were removed, and the hydrogen atoms were added. Then, PXR and the ligand were refined with CHARMM. The active sites of PXR were defined automatically according to the internal ligand binding site through a series of algorithms by DS2.5. A three-dimensional pharmacophore model of the ligand with the targeted protein was generated in the CDOCKER module and Libdock module.

### Statistical Analysis

Data are expressed as mean ± SEM. All data were analyzed with GraphPad Prism 5 (GraphPad Software, San Diego, CA) by one-way analysis of variance (ANOVA) for single-variable analysis or two-way ANOVA for two independent variables, followed by Turkey’s multiple comparisons test. Student’s *t*-test was used for paired data.

## Results

### Prediction of Potential Targets and Signaling Pathways of *Salvia miltiorrhiza* Treatment in AKI

**FIGURE 1:**
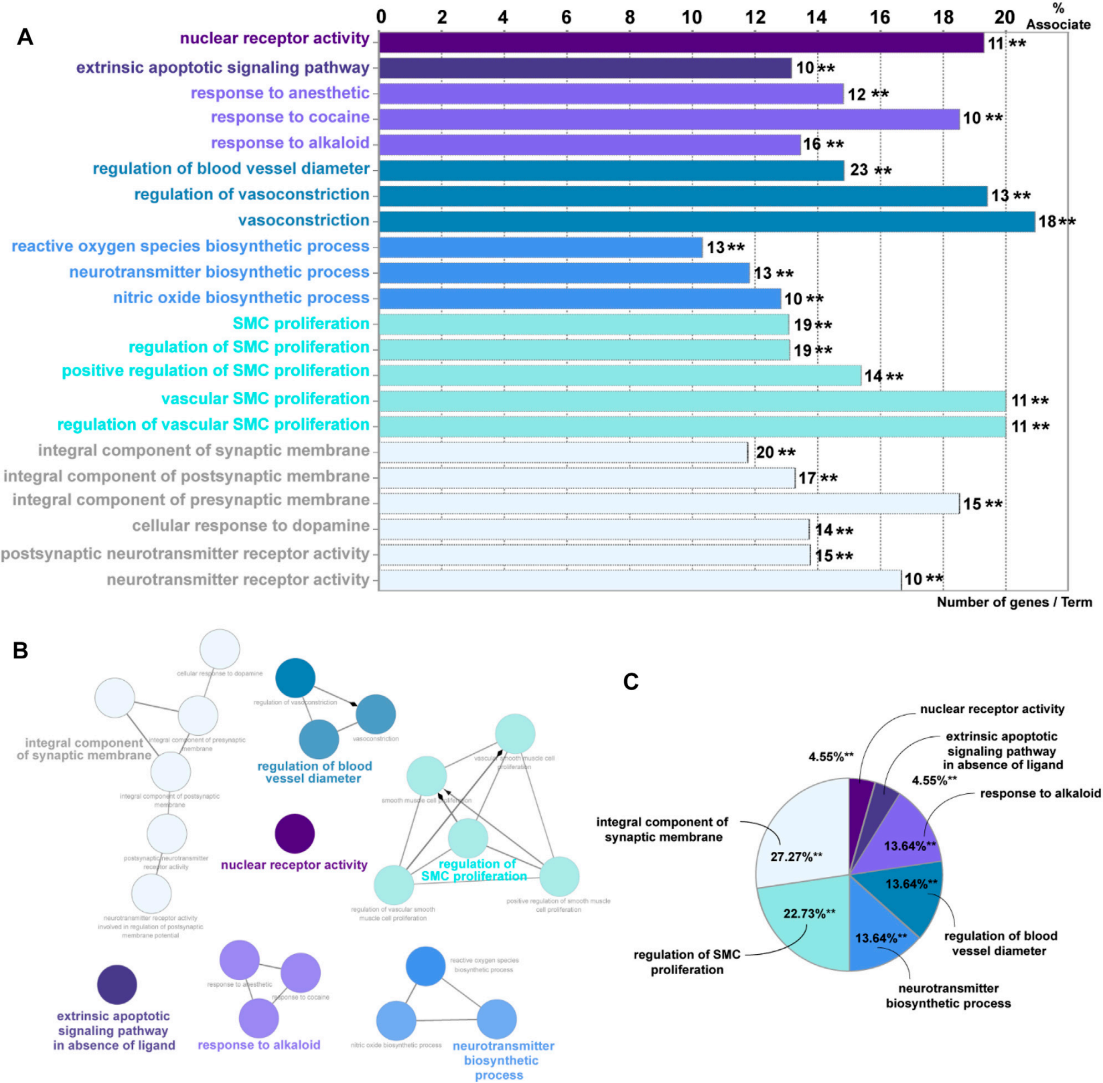
GO enrichment analysis of potential targets and biological networks involved in *Salvia miltiorrhiza* treating AKI. **(A)** Bar chart presenting the percentage of genes involved in different biological functions, and the numbers of related genes in these signaling pathways are intersected. **(B)** Brief view of the 7 GO pathways; all pathways have a *p*-value of < 0.01. **(C)** GO pathways are presented in the pie chart. Detailed information could be found in the supplementary files: Supplementary Table S1: eligible active ingredients; Supplementary Table S2: target proteins involved in the networks; Supplementary Table S3: significant target genes related to the proteins; Supplementary Table S4, Supplementary Figure S1: intersection genes of *Salvia miltiorrhiza* and AKI; Supplementary Figure S2: the KEGG pathways.

Overall, 65 eligible active ingredients (Supplementary Table S1) and 139 target proteins (Supplementary Table S2) were collected from TCMSP. Among these proteins, 133 significant target genes were collected from the String and Uniprot database (Supplementary Table S3); 7123 AKI-related target genes were collected from GeneCards, OMIM, and DisGeNET databases; and 122 intersection genes (Supplementary Table S4, Supplementary Figure S1) of *Salvia miltiorrhiza* and AKI were screened out by the Venn analysis tool. A total of 15 KEGG pathways were enriched, as shown in Supplementary Figure S2. A total of 22 GO pathways were enriched. Figure 1A has indicated the pathways associated with *Salvia miltiorrhiza* treatment in AKI. The numbers of the target genes in the pathway were also highlighted. A pie chart was used to classify 22 GO pathways into 7 significant signaling pathways and percentage of genes related to AKI (as shown in Figure 1B): nuclear receptor activity (4.55%), extrinsic apoptotic signaling pathway (4.55%), response to alkaloid (13.64%), integral component of synaptic membrane (27.27%), regulation of blood vessel diameter (13.64%), regulation of smooth muscle cell proliferation (22.73%), and neurotransmitter biosynthetic process (13.64%) (Figure 1zc C).

Among these pathways, we put great interest in the process of nuclear receptor activity. The nuclear receptor family has shown a percentage of 19.3% association on *Salvia miltiorrhiza* treatment in AKI with 11 target genes involved (AKR1B1, AR, CYP1A1, ESR1, ESR2, NR1I2, NR3C1, PGR, PPARG, RXRA, and STAT3). Up to date, the underlying mechanisms of how nuclear receptors, especially PXR, affect kidney injury are still poorly understood. Following this clue, we tried to reveal the role of PXR in AKI *in vivo* and *in vitro* and seek an alternative possibility in developing *Salvia miltiorrhiza* as a renal protective drug.

### TanIIA Improves Renal Function by Alleviating Renal Injury in Cisplatin-Induced AKI

**FIGURE 2:**
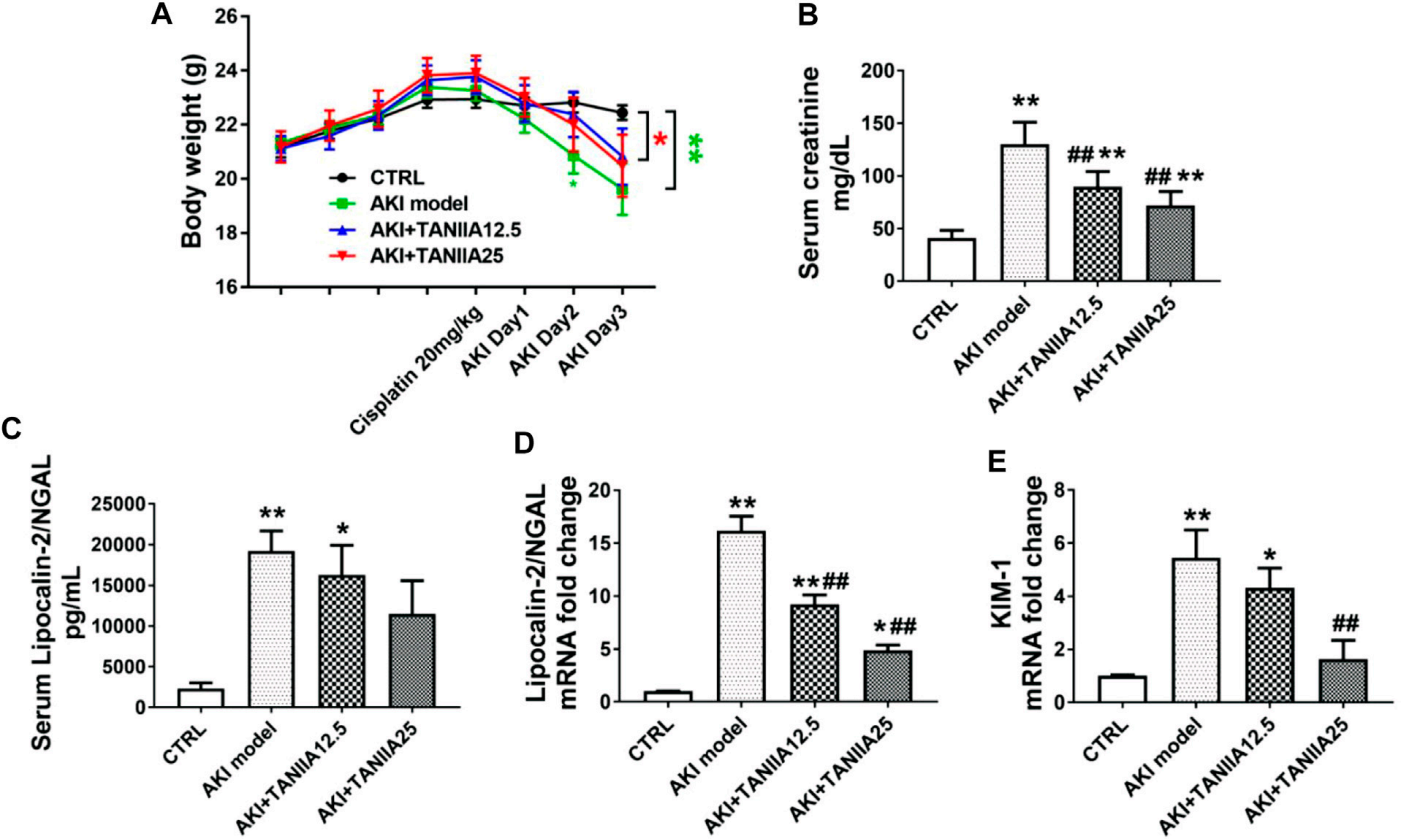
TanIIA treatment restores renal function in cisplatin-induced AKI. **(A)** Body weight (g) before and after cisplatin injection. **(B)** Serum creatinine at 72 h after cisplatin (20 mg/kg) injection. **(C)** Bar chart represents the serum level of NGAL. **(D,E)** mRNA level of NGAL, KIM-1 in the kidney. Data represent as mean ± SEM, *p < 0.05, **p < 0.01 compared with the control group (*n* = 5). *#p* < 0.05, *##p* < 0.01 compared with AKI group (*n* = 5). One-way ANOVA followed by Turkey’s test for multiple comparisons was used for three or more groups; CTRL: 0.9% normal saline; TanIIA concentrations = 12.5 and 25 mg/kg/day; abbreviations: NGAL, neutrophil gelatinase-associated lipocalin; KIM-1, kidney injury molecule 1.

On AKI day 3, the bodyweight of both AKI model and AKI+TanIIA25 (mg/kg/day) was significantly decreased compared to the CRTL group (Figure 2A). As shown in Figure 2B, serum creatinine levels were increased in all cisplatin-treated groups compared to CTRL. However, the creatinine level was reduced in both TanIIA treatment groups compared with the cisplatin-induced AKI model. The neutrophil gelatinase-associated lipocalin (NGAL) and kidney injury molecule-1 (KIM-1) are important biomarkers related to AKI (Schrezenmeier et al., 2017). Consistent with the creatinine results, the serum and mRNA levels of NGAL (Figures 2C,D) and mRNA of KIM-1 were significantly reduced by the TanIIA treatment compared to the AKI model (Figure 2E).

**FIGURE 3:**
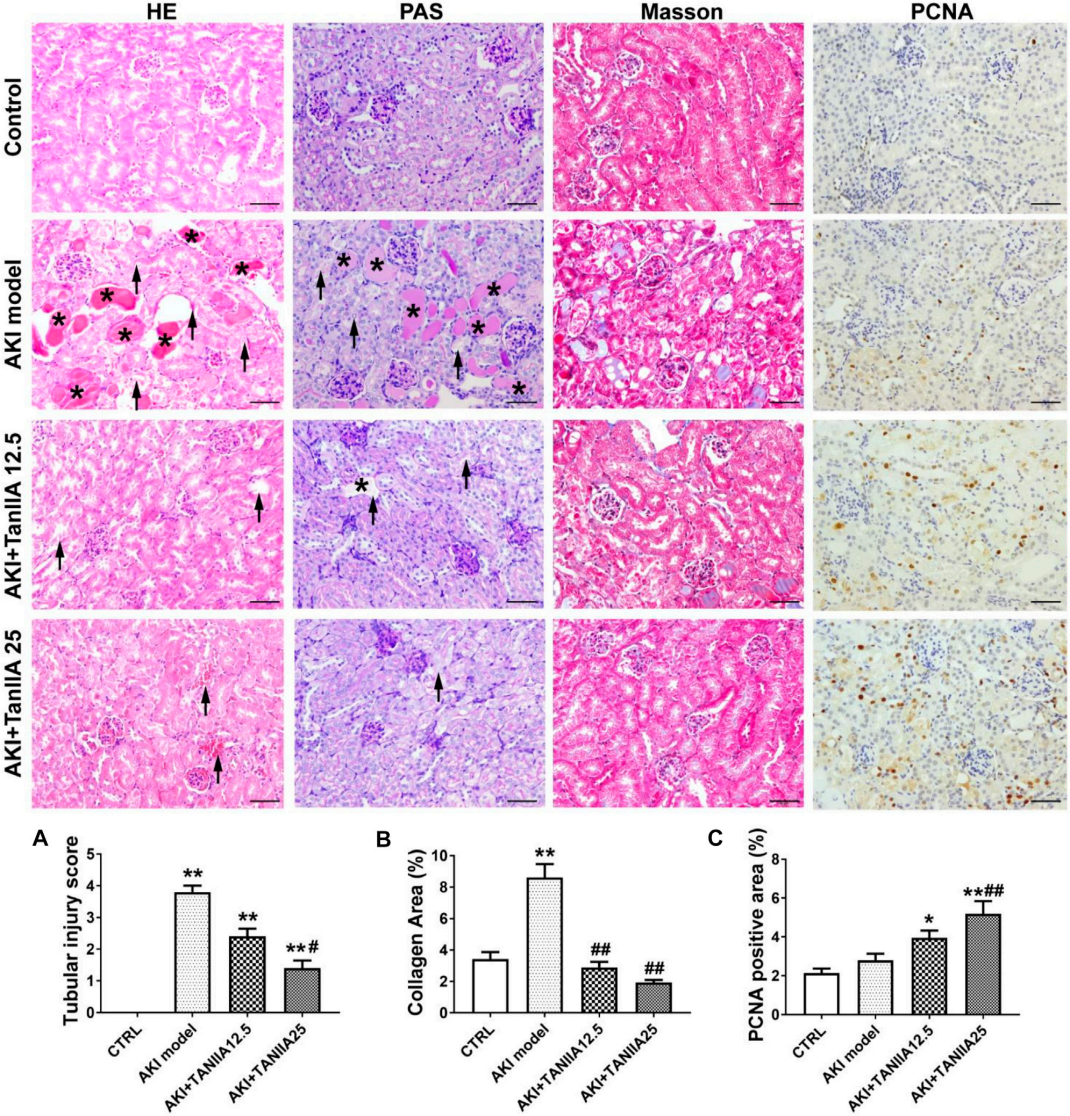
TanIIA improves renal function by preventing cell necrosis and promoting proliferation. **(A,B)** Representative images and relative quantitative data of HE, PAS, and Masson’s trichrome staining in mouse kidney sections. The AKI model group observed tubular necrosis (arrowheads) and casts (asterisks). **(C)** Immunohistochemical staining of PCNA in indicated groups. Data represent as mean ± SEM, *p < 0.05, **p < 0.01 compared with the control group (*n* = 5). *#p* < 0.05, *##p* < 0.01 compared with AKI group (*n* = 5). One-way ANOVA followed by Turkey’s test for multiple comparisons was used for three or more groups. Scale bar, 50 μm; abbreviations: PCNA, proliferating cell nuclear antigen.

Morphological changes of renal damage were shown by HE and PAS staining. Tubular damages such as tubular dilation, atrophy, cast formation, and sloughing of TECs, or loss of the brush border in cisplatin-treated groups were observed (marked as arrowheads and asterisks). TanIIA reduced tubular necrosis, restored the brush border, and formed fewer casts in the tubule, therefore alleviating tubular injuries in a dose-dependent manner compared to the AKI group (Figure 3A). Masson’s trichrome staining indicated an improvement in fibrosis in TanIIA treatment groups, with less collagen formation within the renal interstitial area (Figure 3B). IHC staining has shown an increased staining area of PCNA (cell proliferation marker) in both TanIIA treatment groups compared to the AKI model (Figure 3C).

### TanIIA Activates Increases Arterial Blood Flow and Constrains NF-κB-Induced Renal Inflammation

**FIGURE 4:**
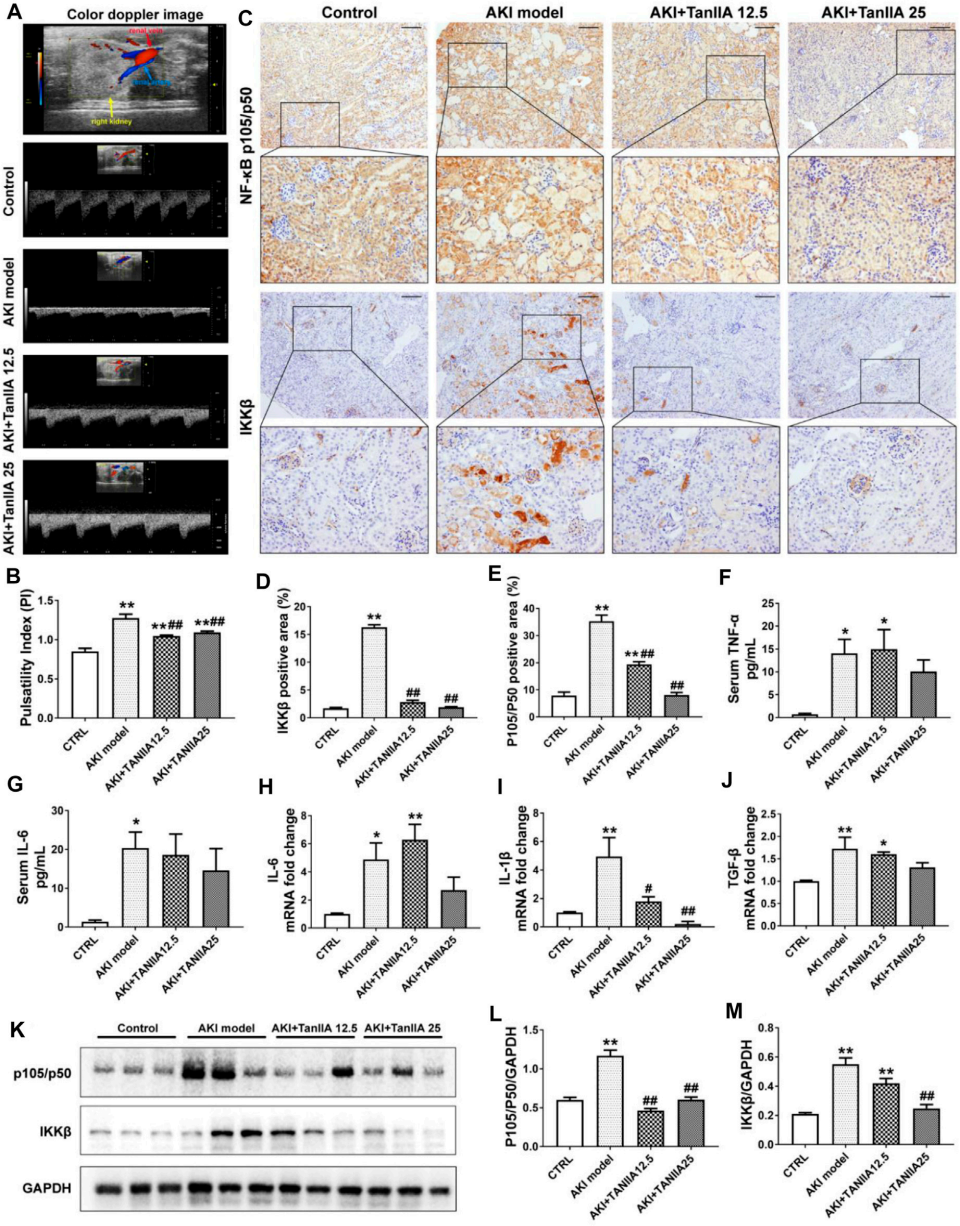
TanIIA improves renal artery blood flow, reduces pro-inflammatory cytokine production, and inhibits the activation of NF-κB signaling. **(A,B)** Representative image of renal vasculature with color Doppler in a mouse kidney (right) and representative bar chart of pulsatility index (PI) in mice. **(C–E)** Immunohistochemical staining of NF-κB p105/p50 and IKKβ and relative quantitative data in indicated groups; **(F,G)** ELISA bar chart representing serum level of TNF-α and IL-6. **(H–J)** mRNA level of IL-6, IL-1β, and TGF-β in the kidney; **(K–M)** Protein expression and relative quantitative data of NF-κB p105/p50 and IKKβ. Data represent as mean ± SEM, *p < 0.05, **p < 0.01 compared with the control group (*n* = 5). *#p* < 0.05, *##p* < 0.01 compared with AKI group (*n* = 5). One-way ANOVA followed by Turkey’s test for multiple comparisons was used for three or more groups. Scale bar, 50 μm; abbreviations: IKKβ, inhibitor of kappa B kinase beta; TNF-α, tumor necrosis factor-alpha; IL-6, interleukin-6; IL-1β, interleukin-1 β; TGF-β, transforming growth factor-β.

It is well-demonstrated that AKI may lead to endothelial dysfunction and impaired vascular autoregulation, which causes vasoconstriction and changes renal hemodynamics. Therefore, the reduction in medullary blood flow can be observed in AKI (Ozkok and Edelstein, 2014). Ultrahigh-frequency ultrasound is a non-invasive and sensitive method that helps monitor renal function. The color Doppler and pulsed wave Doppler images could be used to show renal blood supply and arterial flow velocity patterns (Kelahan et al., 2019). In the present study, renal ultrasonography was used to observe renal blood flow at AKI day 3. The upper panel in Figure 4A has shown the representative image, showing the anatomical relationship of renal vein and artery in normal mouse kidneys. The color Doppler-based sonographic assessment had shown an increase in the pulsatility index (PI) in the AKI model, while PI of the TanIIA treatment was decreased compared to the AKI group (Figure 4B). The results had indicated better renal perfusion in TanIIA-treated mice under AKI conditions.

To investigate how TanIIA affects renal inflammation, we hypothesize that TanIIA inhibits the activation of the NF-κB-activated inflammatory response. Therefore, the expressions of key mediators in NF-κB signaling were measured. The expression of NF-κB p105/p50 and IKKβ was significantly reduced by TanIIA treatment when compared to the AKI model (Figures 4C–E), and the results were confirmed by the NF-κB p105/p50 and IKKβ protein expression measured by Western blot (Figures 4K–M). Similarly, serum levels of pro-inflammatory cytokines in kidneys, such as TNF-α and IL-6, mRNA level of IL-1β, and TGF-β were also declined in TanIIA treatment groups compared to the AKI model (Figures 4F–J). These findings had suggested an anti-inflammatory effect of TanIIA in AKI by inhibiting the production of pro-inflammatory cytokines and the activation of NF-κB signaling.

### TanIIA Human and Mouse PXR and Inhibits PXR-Mediated NF-κB Activity

**FIGURE 5:**
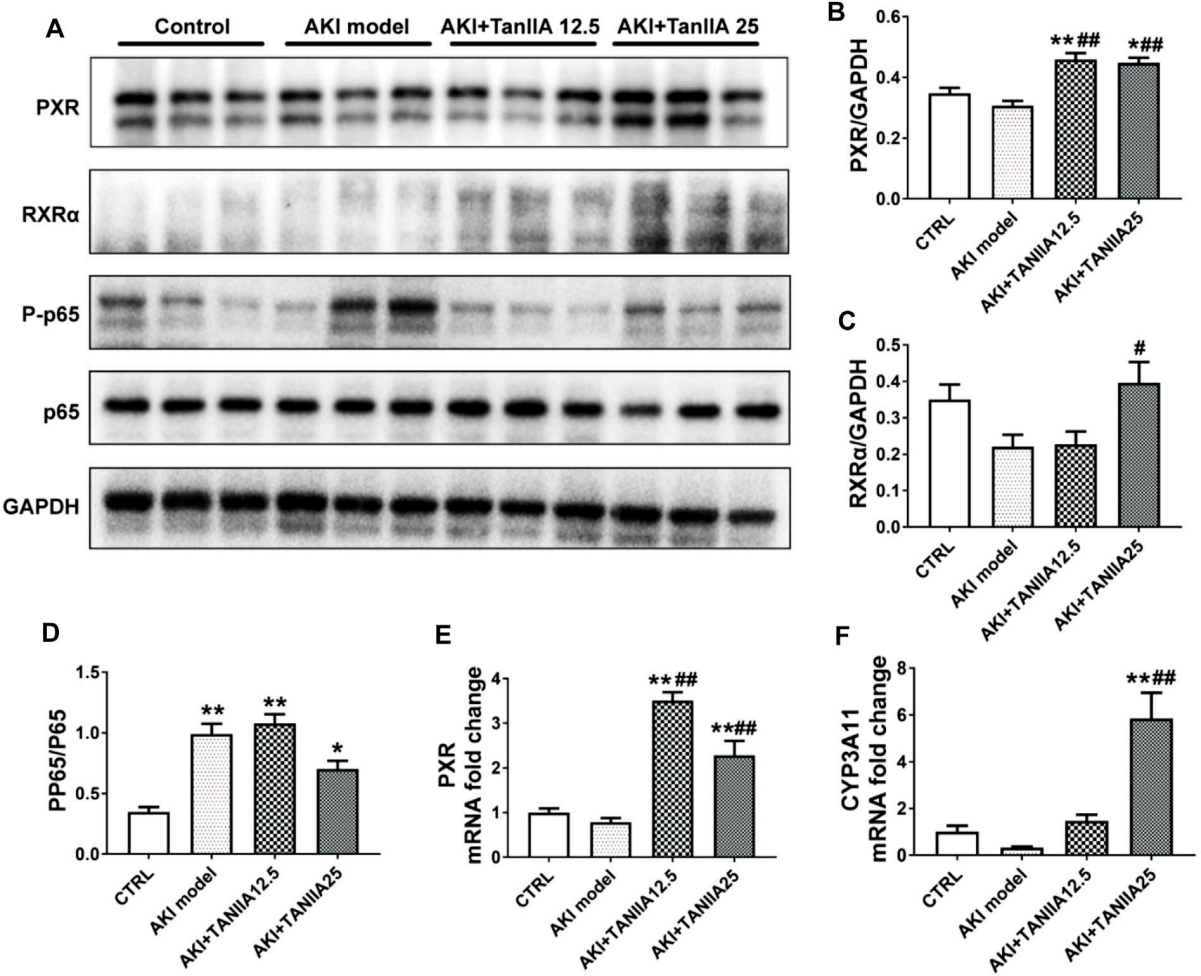
TanIIA actives mouse PXR and inhibit NF-κB activity, and upregulates PXR-mediated *Cyp3a11* expression in AKI model. **(A–D)** The protein expression and relative quantitative data of PXR, RXRα, phospho-NF-κB p65, and NF-κB p65 in kidneys; **(E,F)** mRNA expression of PXR and *Cyp3a11* in kidneys; data represent as mean ± SEM, *p < 0.05, **p < 0.01 compared with the control group (*n* = 5). *#p* < 0.05, *##p* < 0.01 compared with the AKI group (*n* = 5). Student’s *t*-test was used for paired data. One-way ANOVA followed by Turkey’s test for multiple comparisons was used for three or more groups; abbreviations: PP65, phospho-NF-κB p65; P65, NF-κB p65; *Cyp3a11*, cytochrome P450 3A11.

We studied the mechanisms of TanIIA that affected the PXR/NF-κB signaling pathway in AKI mouse and human TEC cell line HK-2. The expression level of mouse PXR and RXRα was significantly decreased in the kidneys by cisplatin treatment (Figures 5A–C). TanIIA treatment significantly induced the expression of mouse PXR, coactivator RXRα, and expression of cytochrome P3A11 (*Cyp3a11*, mouse homolog of CYP3A4). On the other hand, TanIIA suppressed the activation of NF-κB signaling by inhibiting the phosphorylation of NF-κB p65 (Figure 5D). Moreover, reduced mRNA expression of mouse PXR and downstream *Cyp3a11* were also observed in AKI mouse kidneys, while TanIIA treatment upregulated the mRNA expression of mouse PXR and *Cyp3a11* significantly compared to the AKI model (Figures 5E,F).

**FIGURE 6:**
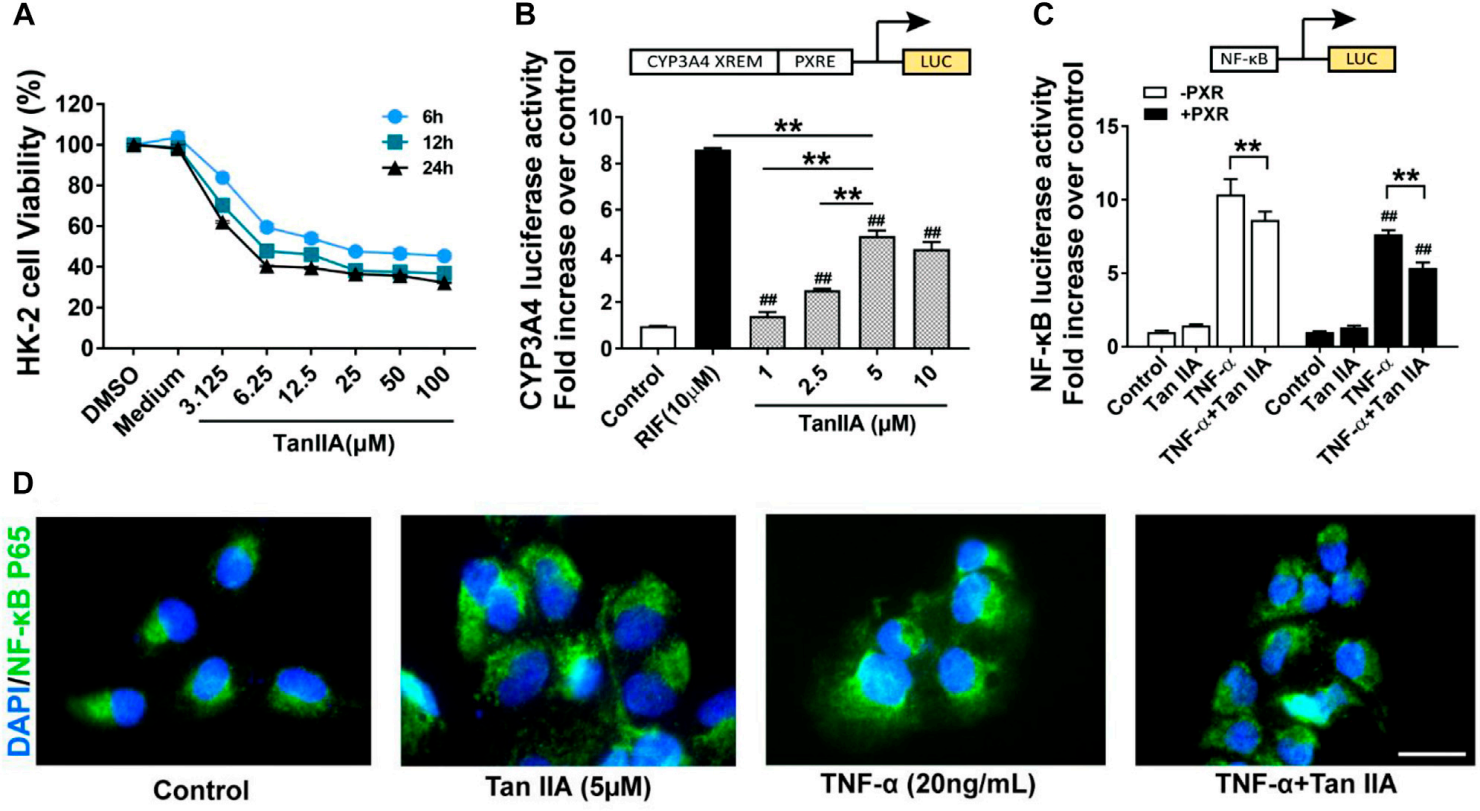
Effects of TanIIA in human PXR activation and human PXR-mediated NF-κB repression. **(A)** Cell viability assay of TanIIA on HK-2 cells; **(B)** TanIIA on PXR-mediated CYP3A4 transcriptional reporter assay. RIF = 10 µM **p < 0.01 compared with indicated groups. *##p* < 0.01 compared with the control group. **(C)** TanIIA on PXR-mediated NF-κB repression reporter assay. HK-2 cells were transfected with/without hPXR small-interfering RNA (siRNA). All cells were transfected with NF-κB luciferase reporter plasmid. HK-2 cells were stimulated by TNF-α (20 ng/ml) to activate NF-κB signaling. **p < 0.01 compared between two groups. #*#p* < 0.01 compared with TNF-α-stimulated cells without human PXR plasmid transfection. **(D)** Immunofluorescence staining images were captured by a fluorescence microscope. Data were represented as mean ± SEM, Student’s *t*-test was used for paired data. One-way ANOVA followed by Turkey’s test for multiple comparisons was used for three or more groups. Scale bar, 25 μm; abbreviations: DMSO, dimethyl sulfoxide; P65, NF-κB p65; RIF, rifampin; TNF-α, tumor necrosis factor α.

A transfection luciferase reporter assay was performed in HK-2 cells to evaluate the effect of TanIIA on human PXR and PXR-mediated CYP3A4 transcription activity. The optimal concentration for TanIIA was chosen by cell viability assay. The IC_50_ values for TanIIA in HK-2 cells were 17.16 ± 1.83 μM (6 h) (Figure 6A). HK-2 cells were transiently transfected with pGL3-CYP3A4-XREM, pSG5-hPXR nuclear receptor expression vector and pRL-TK control vector to investigate whether TanIIA could transcriptionally activate human PXR-mediated CYP3A4 expression. A strong hPXR agonist, rifampin (RIF = 10 µM), was used as the positive control. Cells were incubated with/without TanIIA at different concentrations (TanIIA = 1, 2.5, 5, 10 µM). TanIIA activated human PXR and induced CYP3A4 promoter activity (Figure 6B). We confirmed the optimal concentration of TanIIA (5 µM) to the activation of human PXR.

To explore the role of human PXR on the repression of NF-κB by TanIIA, HK-2 cells were transfected with hPXR small-interfering RNA (siRNA) or the control siRNA. pGL4.32 [luc2P/NF-κB-RE/Hygro], pCMV6-entry or pCMV6-XL4-hPXR, and pRL-TK control vector were transfected into the cells. HK-2 cells were stimulated by TNF-α (20 ng/ml) to activate NF-κB signaling. As shown in Figure 6C, the pro-inflammatory cytokine TNF-α had significantly induced the luciferase activity of NF-κB. When compared to the groups treated with TNF-α alone, TanIIA significantly reduced TNF-α-stimulated NF-κB luciferase expression. In cells treated with TanIIA and stimulated by TNF-α (TNF-α+ TanIIA), NF-κB luciferase activities were significantly reduced in cells cotransfected with human PXR plasmid (pCMV6-XL4-hPXR). The result had suggested TanIIA repressed PXR-mediated NF-κB activity. Besides, in HK-2 cells stimulated by TNF-α, immunofluorescence staining also revealed an inhibitory effect of TanIIA treatment on nuclear translocation of NF-κB p65, suggesting TanIIA alleviated renal inflammation by repressing the activation of NF-κB signaling (Figure 6D).

### Interaction and Binding Mode of TanIIA and PXR

RMSD values are mainly used for analyzing the stability of protein and predicting conformational changes of the protein. RMSD values depend upon the binding interaction and energy between the protein and ligand. The optimized protein has the lowest RMSD values (Carugo and Pongor, 2001). Generally, an RMSD value less than 1Å represents good reproduction of the correct pose. The molecular interactions like hydrogen bonds (H-bonds), hydrophobic interactions (such as π–π interaction and electrostatic interaction), *Van der Waals* interaction, and ionic bonds are also playing an important role in assessing the reliability of results and the binding stability between two modules (Patil et al., 2010).

**FIGURE 7:**
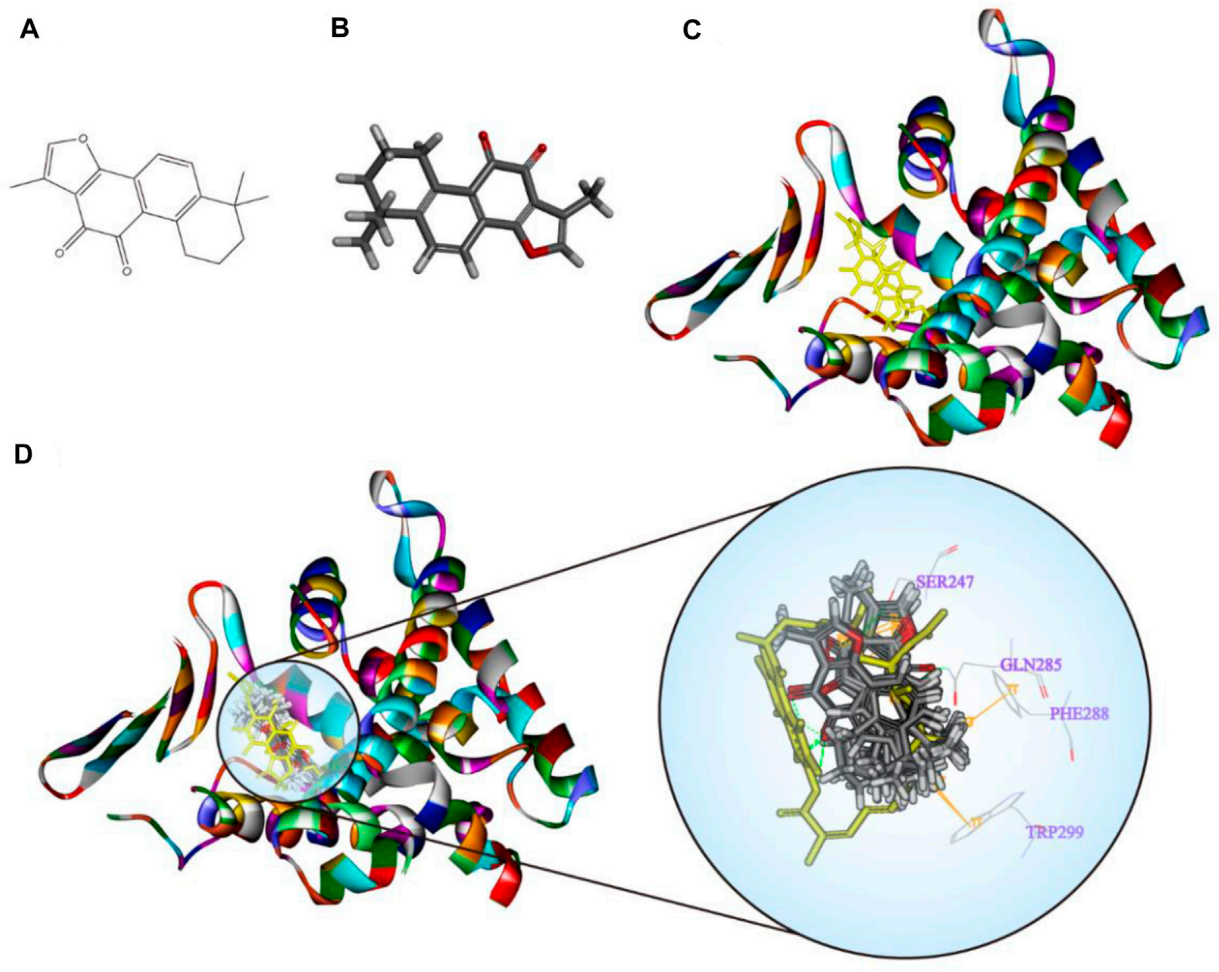
Diagrams of the interaction of TanIIA with the crystal structure of PXR (1SKX). **(A,B)** Chemical and 3D structure of TanIIA; **(C)** the 3D crystal structure of human PXR and ligand-binding domain with an endogenous ligand (yellow) (PDB ID: 1SKX); **(D)** twenty poses of TanIIA docked into the endogenous ligand’s (yellow) active site of 1SKX; the binding model of TanIIA in PXR: at least four residues involved in the interactions in twenty random poses, H-bonds with SER^247^, GLN^285^, and π–π interaction with PHE^288^ and TRP^299^.

TanIIA (chemical and 3D structure are shown in Figures 7A,B) could be docked into the active sites (binding pocket with LBD) of PXR (PDB ID: 1SKX). Inside the binding pocket, PXR consists of an endogenous ligand that had been confirmed as a PXR agonist. This ligand was used as a reference confirmation to compare docked TanIIA (Figure 7C). The RMSD of PXR (PDB ID: 1SKX) was 0.6720 Å (Table 1). Therefore, CDOCKER program was performed to analyze the interactions and binding features of PXR and TanIIA. Inside the binding pocket, TanIIA was surrounded by amino acids of the PXR protein (1SKX). Amino acids SER^247^ and GLN^285^ have formed H-bonds, and PHE^288^ and TRP^299^ formed π–π interactions between TanIIA and PXR (Figure 7D).

**TABLE 1:** Validation of molecular docking algorithm (RMSD).

Protein	CDOCKER RMSD (Å)	Libdock RMSD (Å)
1SKY	0.6720	6.6084

## Discussion

Acute kidney dysfunction impairs the essential physiological homeostasis of the body. Although the kidneys have the remarkable ability to regenerate, progressive inflammation may eventually trigger renal fibrosis and lead to CKD (Gu et al., 2021). *Salvia miltiorrhiza* and its active compounds have been reported to have anti-inflammatory effects in the kidney disease (Gao et al., 2018). However, the role of *Salvia miltiorrhiza* in AKI awaits to be explored.

This study demonstrated the potential therapeutic role of *Salvia miltiorrhiza* and its bioactive compound TanIIA in AKI. Network pharmacological analysis shows a *Salvia miltiorrhiza*-targeted network in AKI, suggesting that the nuclear receptor family could be one of the significant targets. The study using cisplatin-induced AKI mice demonstrates that TanIIA treatment restores renal function in alleviating AKI-induced tubular necrosis and promoting cell proliferation. The production of pro-inflammatory cytokines and interstitial collagen accumulation is reduced by TanIIA treatment. Better perfusion on renal artery blood supply during the acute phase of kidney injury is also observed by color Doppler-based sonographic assessment in AKI mice treated with TanIIA.

Mechanistically, the present study has demonstrated that PXR activation improves renal function in AKI. The finding is consistent with previous findings, Luan *et al.* suggested that PXR activation may protect TECs from apoptosis in AKI by regulating the PI3K/AKT signaling (Luan et al., 2021); Yu *et al.* determined that PXR targeted AKR1B7 to improve mitochondrial metabolism and restored renal function in AKI (Yu et al., 2020). The renal protective effect of TanIIA is dependent on PXR-mediated NF-κB repression. The PXR/NF-κB luciferase reporter assays suggest the role of TanIIA in reducing TNF-α-induced NF-κB activation in a PXR-dependent manner. As for molecular docking, the crystal structure of PXR enables computational investigations on protein–ligand sites and binding modes. We study the entry of TanIIA and its binding inside the PXR cavity. TanIIA stabilizes a binding geometry by forming H-bonds with amino acids SER^247^ and GLN^285^ and forming π–π interactions PHE^288^ and TRP^299^ with PXR. Molecular docking studies have provided evidence on the interaction and binding modes between TanIIA and PXR.

Previous studies have demonstrated a key role of PXR in inflammatory diseases (Ye et al., 2016; Huang et al., 2018; Klepsch et al., 2019). PXR inhibits NF-κB activation to constrain inflammatory response (Deuring et al., 2019; Ren et al., 2019; Okamura et al., 2020). Although substantial PXR-activating xenobiotics have been reported to exert immunosuppressive effects (Wallace et al., 2010; Kittayaruksakul et al., 2013; Zhang J. et al., 2015; Rosette et al., 2019), the human PXR agonist rifampin is not an ideal choice for anti-inflammation treatment due to its adverse effects. The herbal PXR activators such as St. John’s Wort and *Schisandra sphenanthera* (Wuweizi) have been used for a long time which may serve as a beneficial alternative medicine for inflammatory diseases (Mu et al., 2006; Yan et al., 2021). TanIIA is a bioactive compound derived from the root of *Salvia miltiorrhiza* and has been demonstrated as a PXR activator in various diseases (Yu et al., 2009; Zhang et al., 2015a; Zhang et al., 2015b; Zhu et al., 2017; Liu et al., 2020). However, PXR has shown a high degree of cross-species diversity due to the unusually divergent PXR LBD across species. Therefore, PXR of different species may respond diversely to the same ligand. For example, pregnenolone-16a-carbonitrile (PCN) is a potent agonist of mouse PXR but a poor activator of human PXR, and vice versa (Lecluyse, 2001). In this study, TanIIA activates both human and mouse PXR and induces the transcription of their target genes, CYP3A4 and *Cyp3a11*. The mouse model of AKI and NF-κB luciferase reporter assay in human HK-2 cells has indicated that TanIIA inhibits the activation of NF-κB signaling and its target genes. Of note, TanIIA represses both human and mouse PXR-mediated NF-κB activation. These findings are similar to those in the published literature. It is reported that PXR activation constrains the activity of NF-κB signaling that PXR competes with NF-κB for GRIP1 (NF-κB coactivator) interaction (Okamura et al., 2019). Moreover, PXR could directly interact with NF-κB p65, which inhibited the translocation of NF-κB to the nuclear, thereby suppressing the downstream inflammatory response (Wahli, 2008; Zhang et al., 2019; Shao et al., 2021). All these findings have suggested a potential role of PXR/NF-κB signaling in AKI.

Our study has several limitations. We used the C57BL/6 mouse model to establish a cisplatin-induced AKI, instead of the humanized PXR, *Pxr*-null, and wild-type mouse to mimic the human PXR signaling. In addition, we used rifampin as a positive control in HK-2 cells to study how TanIIA affects PXR activity but failed to put PCN as a positive control in the mouse model. Further study is required to provide evidence concerning TanIIA activation to mouse PXR. Due to length limits, we focused on the *Salvia miltiorrhiza*-regulated nuclear receptor family, while other signaling pathways may also serve as potential targets. Further investigation on the renal protective effects of *Salvia miltiorrhiza* is warranted.

## Conclusion

In summary, the present study provides new insights into the renoprotective effect of *Salvia miltiorrhiza* and TanIIA in AKI. TanIIA may ameliorate renal inflammation by repressing PXR-mediated NF-κB activation. The results also suggest TanIIA as a PXR activator against renal inflammation. Our study sheds light on the specific regulatory role of the PXR/NF-κB signaling pathway in kidney pathogenesis, and PXR may serve as a potential target in AKI treatment.

## Data Availability

The datasets presented in this study can be found in online repositories. The names of the repository/repositories and accession number(s) can be found in the article/Supplementary Material.
