# Supplementary material for: Salvia miltiorrhiza Bunge (Danshen) and Bioactive Compound Tanshinone IIA Alleviates Cisplatin-Induced Acute Kidney Injury Through Regulating PXR/NF-κB Signaling
Source: Front Pharmacol. 2022 Mar 24;13:860383. doi: 10.3389/fphar.2022.860383 (PMC8987575; doi:10.3389/fphar.2022.860383)
Supplement: Supplementary file 1 [file DataSheet1.docx]

**Supplementary Data**

**Table S1. Basic information of active compounds in *Salvia miltiorrhiza***

| [**Mol ID**](https://tcmspw.com/tcmspsearch.php?qr=Radix%20Salviae&qsr=herb_en_name&token=f9e6505685e7f20388bc80cca0b93e56) | [**Molecule Name**](https://tcmspw.com/tcmspsearch.php?qr=Radix%20Salviae&qsr=herb_en_name&token=f9e6505685e7f20388bc80cca0b93e56) | [**OB (%)**](https://tcmspw.com/tcmspsearch.php?qr=Radix%20Salviae&qsr=herb_en_name&token=f9e6505685e7f20388bc80cca0b93e56) | [**DL**](https://tcmspw.com/tcmspsearch.php?qr=Radix%20Salviae&qsr=herb_en_name&token=f9e6505685e7f20388bc80cca0b93e56) |
| --- | --- | --- | --- |
| **MOL001601** | [1,2,5,6-tetrahydrotanshinone](https://tcmspw.com/molecule.php?qn=1601) | 38.75 | 0.36 |
| **MOL001659** | [Poriferasterol](https://tcmspw.com/molecule.php?qn=1659) | 43.83 | 0.76 |
| **MOL001771** | [poriferast-5-en-3beta-ol](https://tcmspw.com/molecule.php?qn=1771) | 36.91 | 0.75 |
| **MOL001942** | [isoimperatorin](https://tcmspw.com/molecule.php?qn=1942) | 45.46 | 0.23 |
| **MOL002222** | [sugiol](https://tcmspw.com/molecule.php?qn=2222) | 36.11 | 0.28 |
| **MOL002651** | [Dehydrotanshinone II A](https://tcmspw.com/molecule.php?qn=2651) | 43.76 | 0.4 |
| **MOL002776** | [Baicalin](https://tcmspw.com/molecule.php?qn=2776) | 40.12 | 0.75 |
| **MOL000569** | [digallate](https://tcmspw.com/molecule.php?qn=569) | 61.85 | 0.26 |
| **MOL000006** | [luteolin](https://tcmspw.com/molecule.php?qn=6) | 36.16 | 0.25 |
| **MOL006824** | [α-amyrin](https://tcmspw.com/molecule.php?qn=6824) | 39.51 | 0.76 |
| **MOL007036** | [5,6-dihydroxy-7-isopropyl-1,1-dimethyl-2,3-](https://tcmspw.com/molecule.php?qn=7036)  [dihydrophenanthren-4-one](https://tcmspw.com/molecule.php?qn=7036) | 33.77 | 0.29 |
| **MOL007041** | [2-isopropyl-8-methylphenanthrene-3,4-dione](https://tcmspw.com/molecule.php?qn=7041) | 40.86 | 0.23 |
| **MOL007045** | [3α-hydroxytanshinoneⅡa](https://tcmspw.com/molecule.php?qn=7045) | 44.93 | 0.44 |
| **MOL007048** | [(E)-3-[2-(3,4-dihydroxyphenyl)-7-hydroxy-benzofuran](https://tcmspw.com/molecule.php?qn=7048)  [-4-yl]acrylic acid](https://tcmspw.com/molecule.php?qn=7048) | 48.24 | 0.31 |
| **MOL007049** | [4-methylenemiltirone](https://tcmspw.com/molecule.php?qn=7049) | 34.35 | 0.23 |
| **MOL007050** | [2-(4-hydroxy-3-methoxyphenyl)-5-(3-hydroxypropyl)-](https://tcmspw.com/molecule.php?qn=7050)  [7-methoxy-3-benzofurancarboxaldehyde](https://tcmspw.com/molecule.php?qn=7050) | 62.78 | 0.4 |
| **MOL007051** | [6-o-syringyl-8-o-acetyl shanzhiside methyl ester](https://tcmspw.com/molecule.php?qn=7051) | 46.69 | 0.71 |
| **MOL007058** | [formyltanshinone](https://tcmspw.com/molecule.php?qn=7058) | 73.44 | 0.42 |
| **MOL007059** | [3-beta-Hydroxymethyllenetanshiquinone](https://tcmspw.com/molecule.php?qn=7059) | 32.16 | 0.41 |
| **MOL007061** | [Methylenetanshinquinone](https://tcmspw.com/molecule.php?qn=7061) | 37.07 | 0.36 |
| **MOL007063** | [przewalskin a](https://tcmspw.com/molecule.php?qn=7063) | 37.11 | 0.65 |
| **MOL007064** | [przewalskin b](https://tcmspw.com/molecule.php?qn=7064) | 110.32 | 0.44 |
| **MOL007068** | [Przewaquinone B](https://tcmspw.com/molecule.php?qn=7068) | 62.24 | 0.41 |
| **MOL007069** | [przewaquinone c](https://tcmspw.com/molecule.php?qn=7069) | 55.74 | 0.4 |
| **MOL007070** | [(6S,7R)-6,7-dihydroxy-1,6-dimethyl-8,9-dihydro-7H](https://tcmspw.com/molecule.php?qn=7070)  [-naphtho[8,7-g]benzofuran-10,11-dione](https://tcmspw.com/molecule.php?qn=7070) | 41.31 | 0.45 |
| **MOL007071** | [przewaquinone f](https://tcmspw.com/molecule.php?qn=7071) | 40.31 | 0.46 |
| **MOL007077** | [sclareol](https://tcmspw.com/molecule.php?qn=7077) | 43.67 | 0.21 |
| **MOL007079** | [tanshinaldehyde](https://tcmspw.com/molecule.php?qn=7079) | 52.47 | 0.45 |
| **MOL007081** | [Danshenol B](https://tcmspw.com/molecule.php?qn=7081) | 57.95 | 0.56 |
| **MOL007082** | [Danshenol A](https://tcmspw.com/molecule.php?qn=7082) | 56.97 | 0.52 |
| **MOL007085** | [Salvilenone](https://tcmspw.com/molecule.php?qn=7085) | 30.38 | 0.38 |
| **MOL007088** | [cryptotanshinone](https://tcmspw.com/molecule.php?qn=7088) | 52.34 | 0.4 |
| **MOL007093** | [dan-shexinkum d](https://tcmspw.com/molecule.php?qn=7093) | 38.88 | 0.55 |
| **MOL007094** | [danshenspiroketallactone](https://tcmspw.com/molecule.php?qn=7094) | 50.43 | 0.31 |
| **MOL007098** | [deoxyneocryptotanshinone](https://tcmspw.com/molecule.php?qn=7098) | 49.4 | 0.29 |
| **MOL007100** | [dihydrotanshinlactone](https://tcmspw.com/molecule.php?qn=7100) | 38.68 | 0.32 |
| **MOL007101** | [dihydrotanshinoneⅠ](https://tcmspw.com/molecule.php?qn=7101) | 45.04 | 0.36 |
| **MOL007105** | [epidanshenspiroketallactone](https://tcmspw.com/molecule.php?qn=7105) | 68.27 | 0.31 |
| **MOL007107** | [C09092](https://tcmspw.com/molecule.php?qn=7107) | 36.07 | 0.25 |
| **MOL007108** | [isocryptotanshi-none](https://tcmspw.com/molecule.php?qn=7108) | 54.98 | 0.39 |
| **MOL007111** | [Isotanshinone II](https://tcmspw.com/molecule.php?qn=7111) | 49.92 | 0.4 |
| **MOL007115** | [manool](https://tcmspw.com/molecule.php?qn=7115) | 45.04 | 0.2 |
| **MOL007118** | [microstegiol](https://tcmspw.com/molecule.php?qn=7118) | 39.61 | 0.28 |
| **MOL007119** | [miltionone Ⅰ](https://tcmspw.com/molecule.php?qn=7119) | 49.68 | 0.32 |
| **MOL007120** | [miltionone Ⅱ](https://tcmspw.com/molecule.php?qn=7120) | 71.03 | 0.44 |
| **MOL007121** | [miltipolone](https://tcmspw.com/molecule.php?qn=7121) | 36.56 | 0.37 |
| **MOL007122** | [Miltirone](https://tcmspw.com/molecule.php?qn=7122) | 38.76 | 0.25 |
| **MOL007123** | [miltirone Ⅱ](https://tcmspw.com/molecule.php?qn=7123) | 44.95 | 0.24 |
| **MOL007124** | [neocryptotanshinone ii](https://tcmspw.com/molecule.php?qn=7124) | 39.46 | 0.23 |
| **MOL007125** | [neocryptotanshinone](https://tcmspw.com/molecule.php?qn=7125) | 52.49 | 0.32 |
| **MOL007127** | [1-methyl-8,9-dihydro-7H-naphtho[5,6-g]benzofuran](https://tcmspw.com/molecule.php?qn=7127)  [-6,10,11-trione](https://tcmspw.com/molecule.php?qn=7127) | 34.72 | 0.37 |
| **MOL007130** | [prolithospermic acid](https://tcmspw.com/molecule.php?qn=7130) | 64.37 | 0.31 |
| **MOL007132** | [(2R)-3-(3,4-dihydroxyphenyl)-2-[(Z)-3-](https://tcmspw.com/molecule.php?qn=7132)  [(3,4-dihydroxyphenyl)acryloyl]oxy-propionic acid](https://tcmspw.com/molecule.php?qn=7132) | 109.38 | 0.35 |
| **MOL007140** | [(Z)-3-[2-[(E)-2-(3,4-dihydroxyphenyl)vinyl]-3,4-](https://tcmspw.com/molecule.php?qn=7140)  [dihydroxy-phenyl]acrylic acid](https://tcmspw.com/molecule.php?qn=7140) | 88.54 | 0.26 |
| **MOL007141** | [salvianolic acid g](https://tcmspw.com/molecule.php?qn=7141) | 45.56 | 0.61 |
| **MOL007142** | [salvianolic acid j](https://tcmspw.com/molecule.php?qn=7142) | 43.38 | 0.72 |
| **MOL007143** | [salvilenone Ⅰ](https://tcmspw.com/molecule.php?qn=7143) | 32.43 | 0.23 |
| **MOL007145** | [salviolone](https://tcmspw.com/molecule.php?qn=7145) | 31.72 | 0.24 |
| **MOL007149** | [NSC 122421](https://tcmspw.com/molecule.php?qn=7149) | 34.49 | 0.28 |
| **MOL007150** | [(6S)-6-hydroxy-1-methyl-6-methylol-8,9-dihydro](https://tcmspw.com/molecule.php?qn=7150)  [-7H-naphtho[8,7-g]benzofuran-10,11-quinone](https://tcmspw.com/molecule.php?qn=7150) | 75.39 | 0.46 |
| **MOL007151** | [Tanshindiol B](https://tcmspw.com/molecule.php?qn=7151) | 42.67 | 0.45 |
| **MOL007152** | [Przewaquinone E](https://tcmspw.com/molecule.php?qn=7152) | 42.85 | 0.45 |
| **MOL007154** | [tanshinone iia](https://tcmspw.com/molecule.php?qn=7154) | 49.89 | 0.4 |
| **MOL007155** | [(6S)-6-(hydroxymethyl)-1,6-dimethyl-8,9-dihydro](https://tcmspw.com/molecule.php?qn=7155)  [-7H-naphtho[8,7-g]benzofuran-10,11-dione](https://tcmspw.com/molecule.php?qn=7155) | 65.26 | 0.45 |
| **MOL007156** | [tanshinone Ⅵ](https://tcmspw.com/molecule.php?qn=7156) | 45.64 | 0.3 |

Searched for the keyword “Danshen” in the Traditional Chinese Medicine Systems Pharmacology Database and Analysis Platform (TCMSP [http://tcmspw.com/tcmsp.php)](http://lsp.nwu.edu.cn/index.ph)) to obtain its active ingredients and its ADME (adsorption, distribution, metabolism, and excretion) information (including OB(oral bioavailability), DL(drug likeness), etc.). According to the criteria of OB ≥ 30% and DL ≥ 0.18, the active ingredients that could be well absorbed into the blood were screened as potential active ingredients.

**Table S2. The target proteins of *Salvia miltiorrhiza* screened from TCMSP database**

| **Num** | **Target proteins** | **Num** | **Target proteins** |
| --- | --- | --- | --- |
| 1 | [5-hydroxytryptamine 1A receptor](https://tcmspw.com/target.php?qt=106) | 71 | [Glycogen synthase kinase-3 beta](https://tcmspw.com/target.php?qt=422) |
| 2 | [5-hydroxytryptamine 1B receptor](https://tcmspw.com/target.php?qt=310) | 72 | [Heat shock protein HSP 90](https://tcmspw.com/target.php?qt=444) |
| 3 | [5-hydroxytryptamine 2A receptor](https://tcmspw.com/target.php?qt=175) | 73 | [Heme oxygenase 1](https://tcmspw.com/target.php?qt=2132) |
| 4 | [5-hydroxytryptamine 2C receptor](https://tcmspw.com/target.php?qt=203) | 74 | [Hepatocyte growth factor receptor](https://tcmspw.com/target.php?qt=349) |
| 5 | [5-hydroxytryptamine receptor 3A](https://tcmspw.com/target.php?qt=113) | 75 | [Ig gamma-1 chain C region](https://tcmspw.com/target.php?qt=1201) |
| 6 | [72 kDa type IV collagenase](https://tcmspw.com/target.php?qt=238) | 76 | [Induced myeloid leukemia cell differentiation protein Mcl-1](https://tcmspw.com/target.php?qt=4269) |
| 7 | [Acetylcholinesterase](https://tcmspw.com/target.php?qt=165) | 77 | [Insulin receptor](https://tcmspw.com/target.php?qt=11) |
| 8 | [Activator of 90 kDa heat shock protein ATPase homolog 1](https://tcmspw.com/target.php?qt=4007) | 78 | [Integrin beta-3](https://tcmspw.com/target.php?qt=459) |
| 9 | [Adenylate cyclase type 2](https://tcmspw.com/target.php?qt=4015) | 79 | [Intercellular adhesion molecule 1](https://tcmspw.com/target.php?qt=4287) |
| 10 | [Aldose reductase](https://tcmspw.com/target.php?qt=288) | 80 | [Interferon gamma](https://tcmspw.com/target.php?qt=365) |
| 11 | [Alpha-1A adrenergic receptor](https://tcmspw.com/target.php?qt=191) | 81 | [Interleukin-10](https://tcmspw.com/target.php?qt=4292) |
| 12 | [Alpha-1B adrenergic receptor](https://tcmspw.com/target.php?qt=216) | 82 | [Interleukin-2](https://tcmspw.com/target.php?qt=3978) |
| 13 | [Alpha-1D adrenergic receptor](https://tcmspw.com/target.php?qt=272) | 83 | [Interleukin-4](https://tcmspw.com/target.php?qt=4301) |
| 14 | [Alpha-2A adrenergic receptor](https://tcmspw.com/target.php?qt=105) | 84 | [Interleukin-6](https://tcmspw.com/target.php?qt=351) |
| 15 | [Alpha-2B adrenergic receptor](https://tcmspw.com/target.php?qt=214) | 85 | [Interstitial collagenase](https://tcmspw.com/target.php?qt=353) |
| 16 | [Alpha-2C adrenergic receptor](https://tcmspw.com/target.php?qt=126) | 86 | [Kinetochore protein Nuf2](https://tcmspw.com/target.php?qt=4311) |
| 17 | [Amyloid beta A4 protein](https://tcmspw.com/target.php?qt=648) | 87 | [Matrix metalloproteinase-9](https://tcmspw.com/target.php?qt=4334) |
| 18 | [Androgen receptor](https://tcmspw.com/target.php?qt=48) | 88 | [Mineralocorticoid receptor](https://tcmspw.com/target.php?qt=252) |
| 19 | [Apoptosis regulator Bcl-2](https://tcmspw.com/target.php?qt=86) | 89 | [Mitogen-activated protein kinase 1](https://tcmspw.com/target.php?qt=354) |
| 20 | [Baculoviral IAP repeat-containing protein 4](https://tcmspw.com/target.php?qt=4043) | 90 | [Mitogen-activated protein kinase 14](https://tcmspw.com/target.php?qt=402) |
| 21 | [Baculoviral IAP repeat-containing protein 5](https://tcmspw.com/target.php?qt=4044) | 91 | [mRNA of PKA Catalytic Subunit C-alpha](https://tcmspw.com/target.php?qt=699) |
| 22 | [Bcl-2-like protein 1](https://tcmspw.com/target.php?qt=4054) | 92 | [mRNA of Protein-tyrosine phosphatase, non-receptor type 1](https://tcmspw.com/target.php?qt=229) |
| 23 | [Beta-2 adrenergic receptor](https://tcmspw.com/target.php?qt=261) | 93 | [Muscarinic acetylcholine receptor M1](https://tcmspw.com/target.php?qt=38) |
| 24 | [Beta-lactamase](https://tcmspw.com/target.php?qt=499) | 94 | [Muscarinic acetylcholine receptor M2](https://tcmspw.com/target.php?qt=210) |
| 25 | [Calcitonin receptor](https://tcmspw.com/target.php?qt=168) | 95 | [Muscarinic acetylcholine receptor M3](https://tcmspw.com/target.php?qt=16) |
| 26 | [Calcium-activated potassium channel subunit alpha 1](https://tcmspw.com/target.php?qt=3727) | 96 | [Muscarinic acetylcholine receptor M4](https://tcmspw.com/target.php?qt=154) |
| 27 | [Calmodulin](https://tcmspw.com/target.php?qt=3907) | 97 | [Muscarinic acetylcholine receptor M5](https://tcmspw.com/target.php?qt=87) |
| 28 | [Carbonic anhydrase II](https://tcmspw.com/target.php?qt=117) | 98 | [Mu-type opioid receptor](https://tcmspw.com/target.php?qt=299) |
| 29 | [Caspase-3](https://tcmspw.com/target.php?qt=4087) | 99 | [Myc proto-oncogene protein](https://tcmspw.com/target.php?qt=4372) |
| 30 | [Caspase-7](https://tcmspw.com/target.php?qt=3575) | 100 | [Neuronal acetylcholine receptor protein, alpha-7 chain](https://tcmspw.com/target.php?qt=581) |
| 31 | [Caspase-9](https://tcmspw.com/target.php?qt=4090) | 101 | [Neuronal acetylcholine receptor subunit alpha-2](https://tcmspw.com/target.php?qt=284) |
| 32 | [CD40 ligand](https://tcmspw.com/target.php?qt=4107) | 102 | [NF-kappa-B inhibitor alpha](https://tcmspw.com/target.php?qt=4394) |
| 33 | [Cell division protein kinase 2](https://tcmspw.com/target.php?qt=482) | 103 | [Nitric oxide synthase, inducible](https://tcmspw.com/target.php?qt=3) |
| 34 | [Cell division protein kinase 4](https://tcmspw.com/target.php?qt=573) | 104 | [Nitric-oxide synthase, endothelial](https://tcmspw.com/target.php?qt=95) |
| 35 | [Cellular tumor antigen p53](https://tcmspw.com/target.php?qt=646) | 105 | [Nuclear receptor coactivator 1](https://tcmspw.com/target.php?qt=3279) |
| 36 | [CGMP-inhibited 3',5'-cyclic phosphodiesterase A](https://tcmspw.com/target.php?qt=172) | 106 | [Nuclear receptor coactivator 2](https://tcmspw.com/target.php?qt=3276) |
| 37 | [Coagulation factor VII](https://tcmspw.com/target.php?qt=123) | 107 | [Nuclear receptor subfamily 1 group I member 2](https://tcmspw.com/target.php?qt=4403) |
| 38 | [Coagulation factor Xa](https://tcmspw.com/target.php?qt=79) | 108 | [Nucleophosmin](https://tcmspw.com/target.php?qt=4406) |
| 39 | [Cyclin-A2](https://tcmspw.com/target.php?qt=3025) | 109 | [Peroxisome proliferator activated receptor gamma](https://tcmspw.com/target.php?qt=78) |
| 40 | [Cyclin-dependent kinase inhibitor 1](https://tcmspw.com/target.php?qt=4141) | 110 | [Peroxisome proliferator-activated receptor gamma](https://tcmspw.com/target.php?qt=4422) |
| 41 | [Cytochrome P450 1A1](https://tcmspw.com/target.php?qt=4149) | 111 | [Phosphatidylinositol-4,5-bisphosphate 3-kinase catalytic subunit, gamma isoform](https://tcmspw.com/target.php?qt=491) |
| 42 | [Cytochrome P450 1A2](https://tcmspw.com/target.php?qt=724) | 112 | [Poly [ADP-ribose] polymerase 4](https://tcmspw.com/target.php?qt=4436) |
| 43 | [Cytochrome P450 3A4](https://tcmspw.com/target.php?qt=621) | 113 | [Potassium voltage-gated channel subfamily H member 2](https://tcmspw.com/target.php?qt=37) |
| 44 | [D(1B) dopamine receptor](https://tcmspw.com/target.php?qt=56) | 114 | [Progesterone receptor](https://tcmspw.com/target.php?qt=209) |
| 45 | [D(2) dopamine receptor](https://tcmspw.com/target.php?qt=292) | 115 | [Proliferating cell nuclear antigen](https://tcmspw.com/target.php?qt=4450) |
| 46 | [Delta-type opioid receptor](https://tcmspw.com/target.php?qt=163) | 116 | [Prostaglandin E synthase](https://tcmspw.com/target.php?qt=4456) |
| 47 | [Dipeptidyl peptidase IV](https://tcmspw.com/target.php?qt=332) | 117 | [Prostaglandin G/H synthase 1](https://tcmspw.com/target.php?qt=6) |
| 48 | [DNA topoisomerase 1](https://tcmspw.com/target.php?qt=1293) | 118 | [Prostaglandin G/H synthase 2](https://tcmspw.com/target.php?qt=94) |
| 49 | [DNA topoisomerase 2-alpha](https://tcmspw.com/target.php?qt=4172) | 119 | [Proto-oncogene c-Fos](https://tcmspw.com/target.php?qt=4478) |
| 50 | [DNA topoisomerase II](https://tcmspw.com/target.php?qt=287) | 120 | [Proto-oncogene serine/threonine-protein kinase Pim-1](https://tcmspw.com/target.php?qt=2966) |
| 51 | [Dopamine D1 receptor](https://tcmspw.com/target.php?qt=7) | 121 | [RAC-alpha serine/threonine-protein kinase](https://tcmspw.com/target.php?qt=4490) |
| 52 | [E3 ubiquitin-protein ligase Mdm2](https://tcmspw.com/target.php?qt=4179) | 122 | [Receptor tyrosine-protein kinase erbB-2](https://tcmspw.com/target.php?qt=4496) |
| 53 | [Endothelin-1](https://tcmspw.com/target.php?qt=4187) | 123 | [Retinoblastoma-associated protein](https://tcmspw.com/target.php?qt=2915) |
| 54 | [Endothelin-1 receptor](https://tcmspw.com/target.php?qt=199) | 124 | [Retinoic acid receptor RXR-alpha](https://tcmspw.com/target.php?qt=158) |
| 55 | [Endothelin-converting enzyme 1](https://tcmspw.com/target.php?qt=706) | 125 | [Serine/threonine-protein kinase Chk1](https://tcmspw.com/target.php?qt=647) |
| 56 | [Epidermal growth factor receptor](https://tcmspw.com/target.php?qt=298) | 126 | [Signal transducer and activator of transcription 3](https://tcmspw.com/target.php?qt=4526) |
| 57 | [Estrogen receptor](https://tcmspw.com/target.php?qt=46) | 127 | [Sodium channel protein type 5 subunit alpha](https://tcmspw.com/target.php?qt=70) |
| 58 | [Estrogen receptor beta](https://tcmspw.com/target.php?qt=307) | 128 | [Sodium-dependent dopamine transporter](https://tcmspw.com/target.php?qt=239) |
| 59 | [Fatty acid synthase](https://tcmspw.com/target.php?qt=374) | 129 | [Sodium-dependent noradrenaline transporter](https://tcmspw.com/target.php?qt=186) |
| 60 | [G1/S-specific cyclin-D1](https://tcmspw.com/target.php?qt=4214) | 130 | [Sodium-dependent serotonin transporter](https://tcmspw.com/target.php?qt=290) |
| 61 | [G2/mitotic-specific cyclin-B1](https://tcmspw.com/target.php?qt=4219) | 131 | [Solute carrier family 2, facilitated glucose transporter member 4](https://tcmspw.com/target.php?qt=4535) |
| 62 | [Gamma-aminobutyric acid receptor subunit alpha-1](https://tcmspw.com/target.php?qt=309) | 132 | [Thrombin](https://tcmspw.com/target.php?qt=17) |
| 63 | [Gamma-aminobutyric acid receptor subunit epsilon](https://tcmspw.com/target.php?qt=3967) | 133 | [Transcription factor AP-1](https://tcmspw.com/target.php?qt=414) |
| 64 | [Gamma-aminobutyric acid receptor subunit gamma-3](https://tcmspw.com/target.php?qt=3412) | 134 | [Transcription factor p65](https://tcmspw.com/target.php?qt=4565) |
| 65 | [Gamma-aminobutyric-acid receptor alpha-2 subunit](https://tcmspw.com/target.php?qt=141) | 135 | [Trypsin-1](https://tcmspw.com/target.php?qt=2928) |
| 66 | [Gamma-aminobutyric-acid receptor alpha-3 subunit](https://tcmspw.com/target.php?qt=200) | 136 | [Tumor necrosis factor](https://tcmspw.com/target.php?qt=265) |
| 67 | [Gamma-aminobutyric-acid receptor alpha-5 subunit](https://tcmspw.com/target.php?qt=181) | 137 | [Tyrosinase](https://tcmspw.com/target.php?qt=4590) |
| 68 | [Gamma-aminobutyric-acid receptor subunit alpha-6](https://tcmspw.com/target.php?qt=3284) | 138 | [Vascular endothelial growth factor A](https://tcmspw.com/target.php?qt=740) |
| 69 | [Glucocorticoid receptor](https://tcmspw.com/target.php?qt=308) | 139 | [Xanthine dehydrogenase/oxidase](https://tcmspw.com/target.php?qt=568) |
| 70 | [Glutathione S-transferase P](https://tcmspw.com/target.php?qt=733) |  |  |

**Table S3. The target genes of *Salvia miltiorrhiza* from String and UniProt database**

| **No** | **Gene Symbol** | **No** | **Gene Symbol** | **No** | **Gene Symbol** |
| --- | --- | --- | --- | --- | --- |
| **1** | HTR1A | **45** | TOP1 | **89** | MYC |
| **2** | HTR1B | **46** | TOP2A | **90** | CHRNA2 |
| **3** | HTR2A | **47** | TOP2B | **91** | NFKBIA |
| **4** | HTR2C | **48** | DRD1 | **92** | NOS2 |
| **5** | HTR3A | **49** | MDM2 | **93** | NCOA1 |
| **6** | MMP2 | **50** | EDN1 | **94** | NCOA2 |
| **7** | ACHE | **51** | EDNRA | **95** | NR1I2 |
| **8** | AHSA1 | **52** | ECE1 | **96** | NPM1 |
| **9** | ADCY2 | **53** | EGFR | **97** | PPARG |
| **10** | AKR1B1 | **54** | ESR1 | **98** | PARP4 |
| **11** | ADRA1D | **55** | ESR2 | **99** | KCNH2 |
| **12** | ADRA1B | **56** | FASN | **100** | PGR |
| **13** | ADRA2A | **57** | CCND1 | **101** | PCNA |
| **14** | ADRA2B | **58** | CCNB1 | **102** | PTGES |
| **15** | ADRA2C | **59** | GABRA1 | **103** | PTGS1 |
| **16** | APBA3 | **60** | GABRE | **104** | PTGS2 |
| **17** | AR | **61** | GABRG3 | **105** | FOS |
| **18** | BCL2 | **62** | NR3C1 | **106** | AKT1 |
| **19** | XIAP | **63** | GSTP1 | **107** | ERBB2 |
| **20** | BIRC5 | **64** | GSK3B | **108** | RB1 |
| **21** | BCL2L1 | **65** | HSP90AA1 | **109** | RXRA |
| **22** | ADRB2 | **66** | HMOX1 | **110** | CHEK1 |
| **23** | LACTBL1 | **67** | MET | **111** | STAT3 |
| **24** | CALCR | **68** | MCL1 | **112** | SCN5A |
| **25** | PCP4 | **69** | INSR | **113** | SLC6A3 |
| **26** | CA2 | **70** | ITGB3 | **114** | SLC6A2 |
| **27** | CASP3 | **71** | ICAM1 | **115** | SLC6A4 |
| **28** | CASP7 | **72** | IFNG | **116** | SLC2A4 |
| **29** | CASP9 | **73** | IL10 | **117** | SERPIND1 |
| **30** | CD40LG | **74** | IL2 | **118** | JUN |
| **31** | CDK2 | **75** | IL4 | **119** | RELA |
| **32** | CDK4 | **76** | IL6 | **120** | PRSS1 |
| **33** | TP53 | **77** | MMP1 | **121** | TYR |
| **34** | PDE3A | **78** | NUF2 | **122** | VEGFA |
| **35** | F7 | **79** | MMP9 | **123** | XDH |
| **36** | CCNA2 | **80** | NR3C2 | **124** | CHRNA7 |
| **37** | CDKN1A | **81** | MAPK1 | **125** | IGHG1 |
| **38** | CYP1A1 | **82** | MAPK14 | **126** | PIK3CG |
| **39** | CYP1A2 | **83** | CHRM1 | **127** | NOS3 |
| **40** | CYP3A4 | **84** | CHRM2 | **128** | PIM1 |
| **41** | DRD5 | **85** | CHRM3 | **129** | KCNMA1 |
| **42** | DRD2 | **86** | CHRM4 | **130** | GABRA2 |
| **43** | OPRD1 | **87** | CHRM5 | **131** | GABRA5 |
| **44** | DPP4 | **88** | OPRM1 | **132** | GABRA3 |
|  |  |  |  | **133** | GABRA6 |

Obtained the corresponding target genes according to the obtained target proteins in the TCMSP database via the String (https://string-db.org/) and Uniprot (https://www.uniprot.org/) databases.

**Table S4. The intersection target genes of *Salvia miltiorrhiza* and AKI**

| **Num** | **Target genes** | **Num** | **Target genes** | **Num** | **Target genes** |
| --- | --- | --- | --- | --- | --- |
| 1 | MMP2 | 42 | STAT3 | 83 | GABRA1 |
| 2 | KCNMA1 | 43 | GABRA2 | 84 | NR3C2 |
| 3 | XDH | 44 | CASP7 | 85 | HMOX1 |
| 4 | EDN1 | 45 | GABRA6 | 86 | MAPK1 |
| 5 | NOS2 | 46 | ACHE | 87 | IL4 |
| 6 | CCNB1 | 47 | SERPIND1 | 88 | AKR1B1 |
| 7 | MET | 48 | MCL1 | 89 | RB1 |
| 8 | BCL2 | 49 | IL6 | 90 | TP53 |
| 9 | F7 | 50 | CASP3 | 91 | CASP9 |
| 10 | CYP1A1 | 51 | CYP1A2 | 92 | NCOA2 |
| 11 | HTR3A | 52 | PIK3CG | 93 | HTR2A |
| 12 | CHRM1 | 53 | IFNG | 94 | ESR2 |
| 13 | GSTP1 | 54 | FOS | 95 | SLC6A3 |
| 14 | XIAP | 55 | OPRD1 | 96 | DRD1 |
| 15 | PARP4 | 56 | TOP2A | 97 | ADRA2C |
| 16 | VEGFA | 57 | RELA | 98 | SLC6A4 |
| 17 | MYC | 58 | SLC2A4 | 99 | FASN |
| 18 | CCNA2 | 59 | NOS3 | 100 | DPP4 |
| 19 | GSK3B | 60 | INSR | 101 | KCNH2 |
| 20 | MMP1 | 61 | AKT1 | 102 | PPARG |
| 21 | HTR1A | 62 | NR1I2 | 103 | PIM1 |
| 22 | IL10 | 63 | JUN | 104 | NR3C1 |
| 23 | MDM2 | 64 | AR | 105 | DRD2 |
| 24 | EGFR | 65 | GABRA3 | 106 | ADRA1B |
| 25 | ERBB2 | 66 | CD40LG | 107 | PCNA |
| 26 | CALCR | 67 | CYP3A4 | 108 | ADRB2 |
| 27 | NCOA1 | 68 | PRSS1 | 109 | HSP90AA1 |
| 28 | CDK2 | 69 | BIRC5 | 110 | HTR1B |
| 29 | PGR | 70 | NPM1 | 111 | ADRA2B |
| 30 | TYR | 71 | ADRA2A | 112 | IL2 |
| 31 | CDKN1A | 72 | EDNRA | 113 | ICAM1 |
| 32 | GABRA5 | 73 | PTGES | 114 | BCL2L1 |
| 33 | CHRM3 | 74 | CHRNA2 | 115 | CA2 |
| 34 | NFKBIA | 75 | ECE1 | 116 | CHRM2 |
| 35 | CHRNA7 | 76 | PTGS2 | 117 | OPRM1 |
| 36 | SLC6A2 | 77 | CCND1 | 118 | ITGB3 |
| 37 | RXRA | 78 | ESR1 | 119 | PTGS1 |
| 38 | HTR2C | 79 | GABRG3 | 120 | SCN5A |
| 39 | DRD5 | 80 | CDK4 | 121 | MMP9 |
| 40 | CHEK1 | 81 | PDE3A | 122 | TOP1 |
| 41 | MAPK14 | 82 | ADRA1D |  |  |

**Table S5. Primer used in real time PCR analysis**

| **Gene** | **Primer Sequence** |
| --- | --- |
| Lcn2 (NGAL) | Forward Primer: 5’-CGCTACTGGATCAGAACATTTG-3’  Reverse Primer: 5’-CTTGCACATTGTAGCTCTGTAC-3’ |
| Havcr1 (KIM-1) | Forward Primer: 5’-AGGAAGACCCACGACTATTTCAACAAG-3’  Reverse Primer: 5’-CCTGAGGATGTCACAGTGCCATTC-3’ |
| Il-6 | Forward Primer: 5’-CTCCCAACAGACCTGTCTATAC-3’  Reverse Primer: 5’-CCATTGCACAACTCTTTTCTCA-3’ |
| Il1b (IL-1β) | Forward Primer: 5’-CACTACAGGCTCCGAGATGAACAAC-3’  Reverse Primer: 5’-TGTCGTTGCTTGGTTCTCCTTGTAC-3’ |
| Tgfb1 (TGF-β) | Forward Primer: 5’-CCAGATCCTGTCCAAACTAAGG-3’  Reverse Primer: 5’-CTCTTTAGCATAGTAGTCCGCT-3’ |
| Nr1i2 (PXR) | Forward Primer: 5’-CGATGTGTCAACCTACATGTTC-3’  Reverse Primer: 5’-CTCAGGATGCACATCTCAAAAG-3’ |
| Cyp3a11 | Forward Primer: 5’-GGCAAGCCTGTTACTATGAAAG-3’  Reverse Primer: 5’-ACTGAGAAGAGCAAAGGATCAA-3’ |
| Actb (β-actin) | Forward primer: 5’-CTACCTCATGAAGATCCTGACC-3’  Reverse Primer: 5’-CACAGCTTCTCTTTGATGTCAC-3’ |


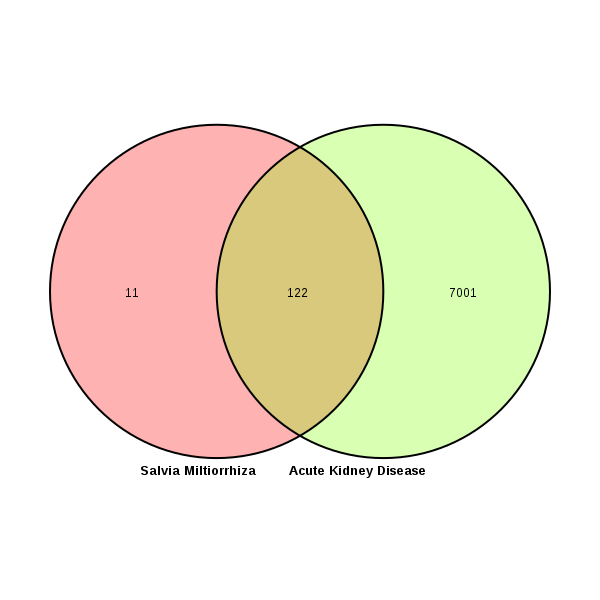


**Figure S1: Venn diagram for the intersection taget genes of *Salvia miltiorrhiza* and AKI.** 7123 target genes of AKI were obtained from GeneCards, OMIM and DisGeNET databases. Venn analysis tool was used to obtain the intersection of target genes of *salvia miltiorrhiza* and AKI, and 122 relevant target genes related to *salvia miltiorrhiza* that may be used to treat AKI were screened out.


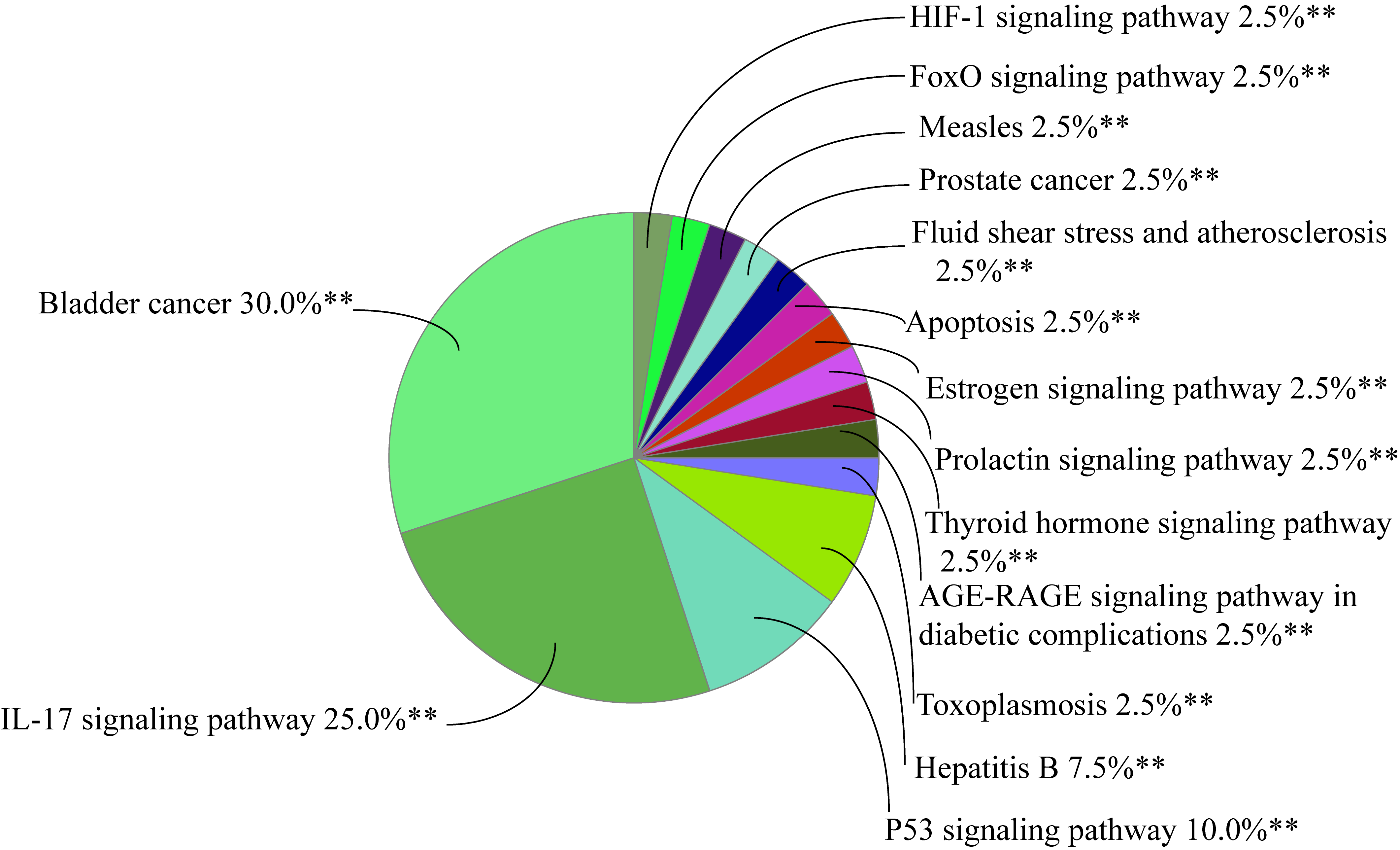


**Figure S2: The KEGG enrichment analysis of potential targets of Salvia miltiorrhiza in treating AKI.**


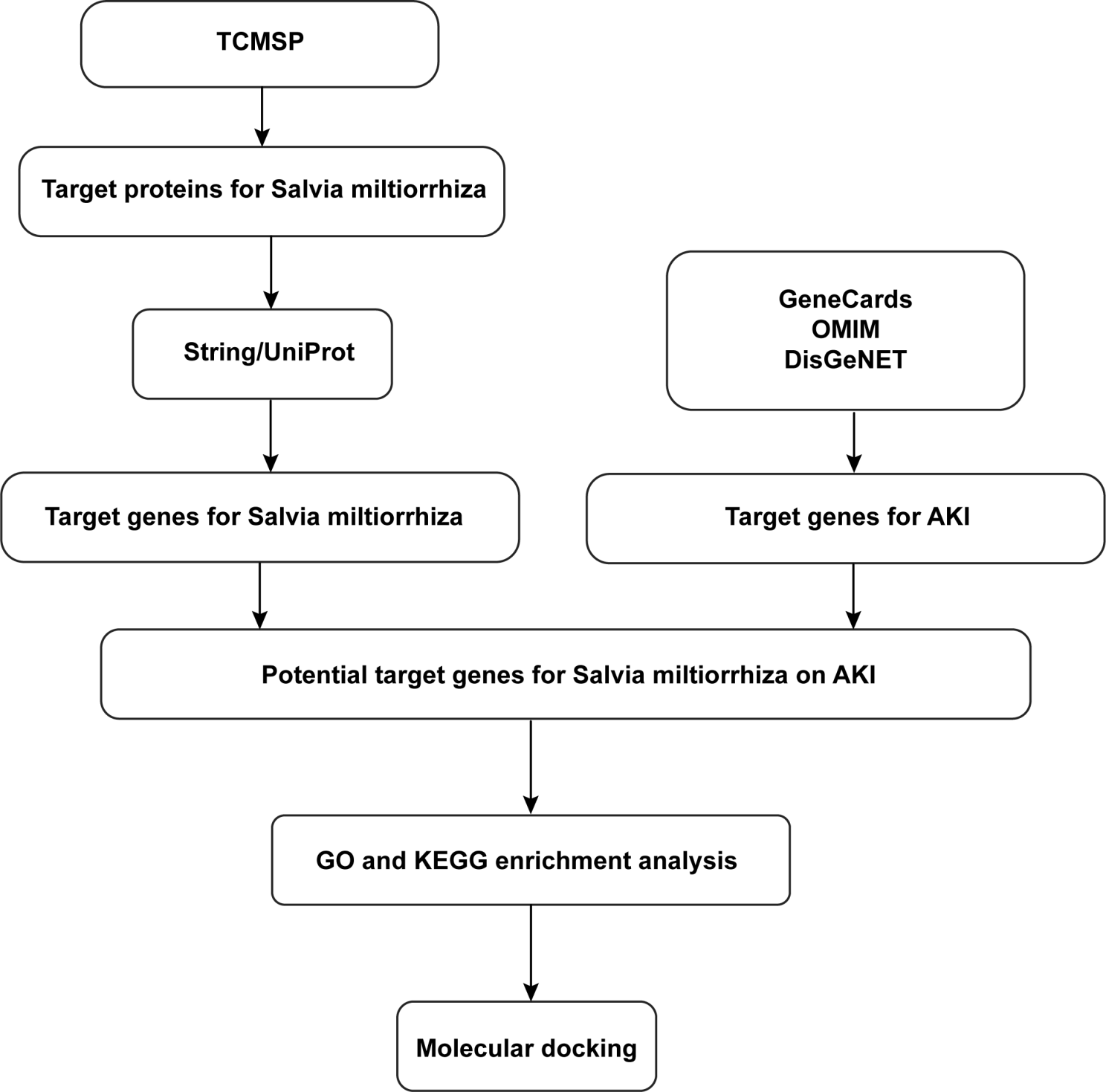


**Figure S3. Flow chart of the network pharmacological analysis of the study.**
